# Microwave Synthesis, Evaluation, and Docking Study of Amino Acid Derivatives of 7‑Chloroquinoline: Exploring Cytotoxic and Antioxidant Potentials

**DOI:** 10.1021/acsomega.5c09882

**Published:** 2026-01-02

**Authors:** James A. Ezugwu, Fatümetüzzehra Küçükbay, Samet Öz, Tuba Keskin, Houssem Boulebd, Suat Tekin, Hasan Küçükbay

**Affiliations:** 1 Department of Pure and Industrial Chemistry, University of Nigeria, Nsukka, 410001 Enugu State, Nigeria; 2 İnönü University, Faculty of Pharmacy, Department of Basic Pharmaceutical Sciences, 44280 Malatya, Turkey; 3 37520 Osmaniye Korkut Ata University, Health Services Vocational School, Laboratory and Veterinary Health Department, 80000 Osmaniye, Turkey; 4 İnönü University, Faculty of Medicine, Department of Physiology, 44280 Malatya, Turkey; 5 Laboratory of Synthesis of Molecules With Biological Interest, Department of Chemistry, Faculty of Exact Sciences, University Frères Mentouri Constantine 1, 25000 Constantine, Algeria; 6 İnönü University, Faculty of Arts and Sciences, Department of Chemistry, 44280 Malatya, Turkey

## Abstract

New carbamate and amino acid derivatives of 7-chloroquinoline were synthesized and characterized using FTIR, ^1^H NMR, ^13^C NMR, and HRMS analysis. The synthesized compounds were obtained through a benzotriazole-mediated approach via microwave synthesis and evaluated for antioxidant and anticancer activities. All the synthesized compounds have antioxidant properties though less than those of the standard. Cytotoxic activities of new 7-chloroquinolinyl benzyl amino carbamate derivatives were accessed using LNCaP (Lymph Node Carcinoma of the Prostate), A2780 (human ovarian cancer), and MCF-7 (breast cancer) cell lines. For cytotoxicity research, the 3-(4,5-dimethylthiazol-2-yl)-2,5-diphenyltetrazolium bromide (MTT) assay was used. The synthesized compounds were subjected to a cell viability assay, and following a 24 h treatment, the IC_50_ values were determined. Among all the tested compounds, compound **4b** demonstrated comparable antitumor activity against LNCaP, A2780, and MCF-7 cell lines when compared to the standard drug docetaxel with IC_50_ values of 6.61, 2.81, and 5.69 μg/mL for LNCaP, A2780, and MCF-7 cell lines, respectively. A molecular docking study targeting the β-tubulin enzyme revealed that compounds **4a**, **4b**, and **5b** exhibit a high affinity for the taxane binding site and may mimic the action of docetaxel.

## Graphical abstract

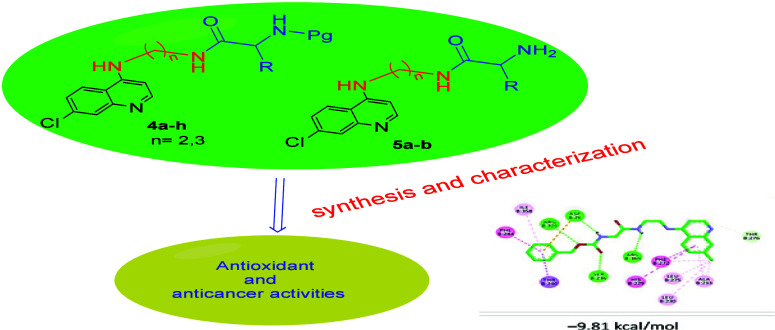

## 1 Introduction

Cancer continues to pose a global health challenge. Many factors such as aging populations, unhealthy behaviors, infections, and environmental exposures contribute to the growing global burden of cancer.^1^ Cancer imposes harmful psychosocial impacts and financial strain on both the general public and health systems.^2^ Twenty million new cases and 9.7 million cancer-related deaths were reported globally in 2022, with nearly 70% of deaths occurring in developing countries.^3^ In addition to ensuring timely and accurate diagnosis, access to appropriate treatments remains a major challenge in improving survival rates across cancer types and ultimately overcoming the disease.^4^ Furthermore, free radicals can damage body cells, contributing to the development of serious conditions such as cancer, cataracts, premature aging, and other degenerative disorders.^5^ Antioxidants play a vital role in preventing several diseases, including cancer, malaria, and neurodegenerative disorders.^6^ Thus, the synthesis of new hybrids with cytotoxic activity remains a foremost goal in organic and medicinal chemistry. 7-Chloroquinoline is an important aromatic heterocyclic pharmacophore comprising fused benzene and pyridine rings. The 7-chloroquinoline scaffold has been utilized for decades in drug design and development. To this day, compounds containing this moiety continue to serve as valuable scaffolds for designing new synthetic agents with diverse biological activities. 7-Chloroquinoline derivatives attract considerable research attention due to their diverse pharmacological and biological activities, including antimalarial,^7^ anticancer,^8,9^ antileishmanial,^9^ antituberculosis,^10^ antimicrobial^11^ and antioxidant properties.^12^ At present, well-known antimalarial 7-chloroquinolines such as chloroquine and hydroxychloroquine have been evaluated in clinical trials as promising anticancer agents.^13,14^

A recent study reported novel 4-aminoquinoline derivatives designed and synthesized using a hybrid pharmacophore strategy to enhance their anticancer potential.^15^

Moreover, a series of new 7-chloroquinoline hydrazones exhibited good antiproliferative activity against several cancer cell lines.^16^ Unlike previous quinoline derivatization routes that relied on stepwise reaction methods, our benzotriazole-mediated microwave protocol offers a greener, single-step synthesis with improved yields and reduced reaction times. This method also allows selective coupling at the primary amine, affording unique carbamate–amino acid hybrids that have not been previously reported. Thus, we envisioned that coupling an amino-acid-bearing carbamate with a 7-chloroquinoline moiety would yield novel derivatives with potential applications in cancer therapy.

In this study, we report the preparation of five Z-protected and three Boc-protected amino acid derivatives bearing a 7-chloroquinoline moiety, along with their two deprotected analogues, and evaluation of their cytotoxic and antioxidant properties.

## 2 Results and Discussion

### 2.1 Synthesis and Characterization of the New Carbamate and Amino Acid Derivatives of 7-Chloroquinoline

The synthesis of novel carbamate and amino acid derivatives of 7-chloroquinoline reported in this study is depicted in Schemes 1–3. A benzotriazole-mediated methodology was adopted to synthesize the desired 7-chloroquinolin-4-yl-aminocarbamate and amino acid hybrids. Compounds **4a**–**h** were obtained via a straightforward benzotriazole-mediated acylation reaction carried out in a single step at 70 °C under microwave irradiation for 1 h in THF, affording the desired products in good yields (Scheme 2). The compounds were purified by crystallization using a DCM/*n*-hexane (1:2) mixture. The protection group in carbamate derivatives of 7-chloroquinoline was transformed to yield the corresponding amino derivatives (Scheme 3), in order to examine the effect of the protecting group. The 7-chloroquinoline–amino acid conjugates, **5a**,**b**, were obtained by treating compounds **4g**,**h** in a TFA/DCM mixture at 25 °C for 1 h. The structures of the products were fully elucidated by ^1^H and ^13^C NMR and MS.

**Scheme 1:**
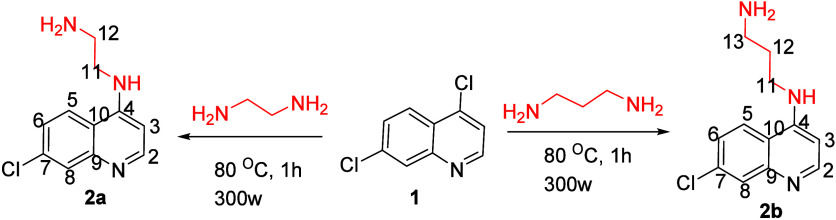
Synthesis of *N* ^1^-(7-Chloroquinolin-4-yl)­alkanediamine (**2a**,**b**)

**Scheme 2:**
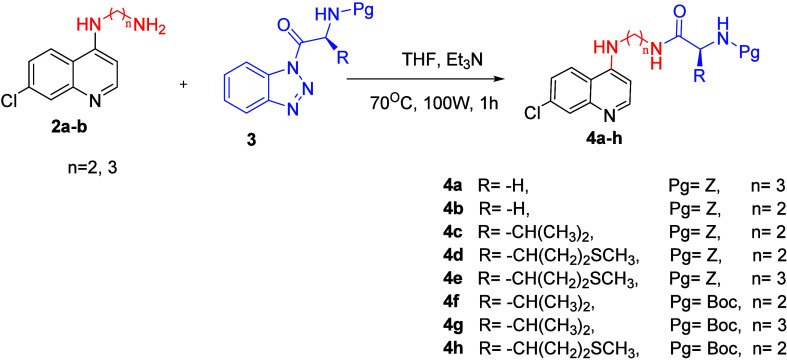
Synthesis of 7-Chloroquinolin-4-yl-aminocarbamate Derivatives (**4a**–**h**)

**Scheme 3:**
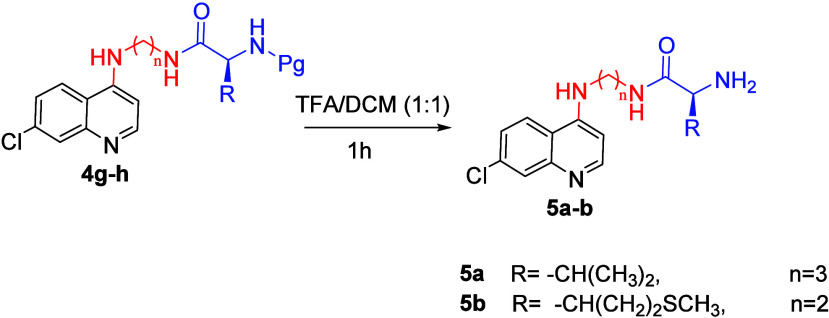
Synthesis of 7-Chloroquinoline–Amino Acid Conjugates (**5a**,**b**)

In the ^1^H NMR spectrum of **4a**, the characteristic NH of amide and other NH resonances of the 7-chloroquinoline part of the synthesized compounds were observed at δ8.01 and δ7.52 ppm regions as triplet peaks, while the NH of the carbamate part of the synthesized compounds was observed in the δ7.79 ppm region as a doublet peak. The aromatic protons were ascertained at δ 8.40, 8.24, 7.48–7.43, 7.36, 7.31, and 6.48 ppm as singlet, doublet, and multiplet peaks. The methylene group of the protecting group displayed a singlet peak at 5.04 ppm, and the methylene group of glycine showed as a doublet at 3.61 ppm. The methylene groups of the 7-chloroquinoline part were observed at 3.60–3.26 ppm, 3.23–3.18 ppm, and 1.81–1.73 ppm as doublet, multiplet, and multiplet peaks, respectively. In the ^13^C NMR spectrum, the carbonyl carbons of the amide group and carbamate group were observed at δ169.7 and 157.0 ppm, respectively, while the aromatic carbon peaks and aliphatic carbon peaks were observed from 99.2 to 152.4 ppm and from 28.3 to 66.0 ppm, respectively. Also, the HRMS peak at *m*/*z* 427.1531 [M + H]^+^ confirmed the formation of **4a**. All of the synthesized compounds were in agreement with their structures.

### 2.2 Biological Activity

#### 2.2.1 Antioxidant Activity

The antioxidant activity of ten synthesized 7-chloroquinolin-4-yl-aminocarbamate derivatives (**4a**–**h**, **5a**,**b**) was evaluated based on their ability to scavenge DPPH free radicals, and the results are presented in Table 1.

**Table 1:** Antioxidant Activities of the Carbamate and Amino Acid Derivatives of 7-Chloroquinoline (**4a**–**h**, **5a**,**b**)

	DPPH Free Radical Scavenging Activity (%)				
Compd No.	12.5 μg/mL	25 μg/mL	37.5 μg/mL	62.5 μg/mL	125 μg/mL
**4a**	5.35	6.25	7.45	8.23	8.28
**4b**	4.55	5.67	7.35	8.55	10.05
**4c**	13.72	26.54	37.88	49.49	50.17
**4d**	4.40	5.25	5.26	5.20	5.20
**4e**	3.56	4.15	4.88	4.95	4.96
**4f**	3.73	3.85	3.87	3.86	3.84
**4g**	3.73	3.85	3.87	3.86	3.84
**4h**	3.45	4.44	6.54	8.07	10.75
**5a**	4.48	6.67	8.45	9.98	10.02
**5b**	3.42	3.66	3.87	4.02	4.40
BHT	58.98	61.01	63.02	67.80	72.34

The standard antioxidant BHT exhibited the highest radical-scavenging activity in a concentration-dependent manner, increasing from 58.98% at 12.5 μg/mL to 72.34% at 125 μg/mL. Among the tested compounds, **4c** displayed the most significant activity, with values ranging from 13.72% at 12.5 μg/mL to 50.17% at 125 μg/mL, thereby demonstrating a strong dose-dependent effect, although still lower than that of the reference standard. Compounds **4b**, **4h**, and **5a** showed moderate activity, reaching ∼10% inhibition at the highest concentration tested, whereas compounds **4a**, **4d**, **4e**, **4f**, **4g**, and **5b** exhibited very weak or negligible scavenging potential (<10%) with nearly flat dose–response curves. In evaluating the effect of protection of the amino group, it was revealed in Table 1 that **5a** exhibited superior antioxidant activity compared with **4g**. While **5a** reached ∼10% scavenging at higher doses, **4g** is almost inactive, plateauing around 3.8%. The enhanced radical-scavenging ability of **5a** compared to its protected analogue **4g** can be attributed to the presence of a free −NH_2_ group, which can donate hydrogen atoms or electrons to quench DPPH radicals. The protection in **4g** blocks this site, thereby reducing its antioxidant capacity. Compound **4h** exhibited much stronger activity than **5b**; at the highest concentration, **4h** (∼10.75%) demonstrated more than double the activity of **5b** (∼4.40%) maybe due to increased polarity and reduced cell permeability. Compound **5b** was essentially inactive, whereas **4h** showed mild but noticeable antioxidant potential. The generally weak DPPH scavenging may arise from the electron-deficient nature of the 7-chloroquinoline ring and steric hindrance of the carbamate linkage, both of which limit hydrogen-donor availability.

#### 2.2.2 Cytotoxicity Studies

The new carbamate and amino acid derivatives of 7-chloroquinoline were evaluated for cytotoxicity using LNCaP (Lymph Node Carcinoma of the Prostate), A2780 (human ovarian cancer), and MCF-7 (breast cancer) cell lines. To determine the cytotoxic properties of the new synthesized compounds, the three cell lines (LNCaP, A2780, and MCF-7) were subjected to an MTT (3-(4,5-dimethylthiazol2-yl)-2,5-diphenyltetrazolium bromide) assay after incubation with the compounds at concentrations ranging from 0.1 to 100 μg/mL for a duration of 24 h. A 24 h time-dependent cell viability assay was carried out through which the IC_50_ values (μg/mL) of the synthesized **4a**–**h** and **5a**,**b** were obtained according to the MTT assay results as presented in Table 2.

**Table 2:** IC_50_ (μg/mL) Concentrations Calculated for LNCaP, A2780, and MCF-7 Cells of New Carbamate and Amino Acid Derivatives of 7-Chloroquinoline, **4a**–**h** and **5a**,**b**

Compound	LNCaP IC_50_ (μg/mL)	A2780 IC_50_ (μg/mL)	MCF-7 IC_50_ (μg/mL)
**4a**	29.48 ± 0.19	9.01 ± 0.23	8.93 ± 0.26
**4b**	6.61 ± 0.09	2.81 ± 0.12	5.69 ± 0.13
**4c**	13.51 ± 0.29	57.1 ± 0.43	89.79 ± 0.57
**4d**	206.1 ± 0.45	78.75 ± 0.31	60.49 ± 0.58
**4e**	18.72 ± 0.18	141.7 ± 0.27	191.6 ± ± 0.09
**4f**	194.7 ± 0.16	10.73 ± 0.12	10.58 ± 0.33
**4g**	48.66 ± 0.08	144.6 ± 0.04	113.5 ± ± 0.16
**4h**	99.81 ± 0.34	116.7 ± 0.34	85.73 ± 0.35
**5a**	9.83 ± 0.02	2.97 ± 0.18	12.89 ± 0.26
**5b**	16.38 ± 0.12	13.68 ± 0.27	24.67 ± 0.18
Docetaxel	0.58 ± 0.24	0.37 ± 0.16	0.56 ± 0.23

According to the data presented in Table 2, **4b** showed the highest antitumor activity against LNCaP, A2780, and MCF-7 cell lines among the synthesized compounds with IC_50_ values of 6.61, 2.81, and 5.69 μg/mL, respectively. Compound **4b** exhibited the highest cytotoxic activity, likely due to its shorter ethylene linker, which allows optimal orientation of the 7-chloroquinoline nucleus and carbamate carbonyl for hydrogen-bonding interactions within the β-tubulin binding pocket. In contrast, compounds with propylene linkers (*n* = 3) such as **4a** and **4g** showed reduced activity, suggesting that excessive flexibility diminishes binding affinity.

The cytotoxicity data revealed a clear structure–activity relationship. The removal of the protecting group from *tert*-butyl carbamate allowed for the synthesis of derivatives containing free amino groups (**5a** and **5b**), which dramatically increased the potency across all three cell lines. These data indicate that the terminal amine is a critical pharmacophore likely mediating H-bond or ionic interactions with the molecular target and/or enhancing cellular uptake. The deprotected derivatives **5a** and **5b** were more active (IC_50_ = 9.831, 2.974, and 12.89 μg/mL and 16.38, 13.68, and 24.67 μg/mL, respectively) than their Boc-protected precursors **4g** and **4h** (IC_50_ = 48.66, 78.75, and 60.49 μg/mL and 99.81, 116.7, and 85.73 μg/mL, respectively).

The cell viability outcomes of LNCaP, A2780, and MCF-7 cells obtained after 24 h treatment with the carbamate and amino acid derivatives of 7-chloroquinoline are presented in Tables 3–5, respectively. The data in Table 3 revealed that all carbamate and amino acid derivatives of 7-chloroquinoline demonstrate cytotoxic activity against the LNCaP cell line at 10 and 100 μg/mL concentrations (*p* < 0.05), apart from **4a**, **4d**, and **4f** at 10 μg/mL concentration. In Table 4, with exception of compound **4f** at10 μg/mL, all compounds possess cytotoxic activity against the A2780 cell line at 10 and 100 μg/mL (*p* < 0.05). Also, in Table 5, only compounds **4d**, **4f**, and **5b** show no cytotoxic activity at 10 μg/mL (*p* > 0.05); all others show activity at 10 and 100 μg/mL.

**Table 3:** LNCaP Cell Viability (%) Results with Compounds **4a**–**h** and **5a**,**b** ^a^

Compd	Control	DMSO	0.1 μg/mL	1 μg/mL	10 μg/mL	100 μg/mL
**4a**	100 ± 9.93	91.42 ± 10.63	96.69 ± 10.23	98.97 ± 8.89	88.16 ± 9.54	7.91 ± 1.12*
**4b**	100 ± 9.93	91.42 ± 10.63	90.43 ± 8.07	92.05 ± 9.87	37.52 ± 3.44*	7.48 ± 0.81*
**4c**	100 ± 9.93	91.42 ± 10.63	73.62 ± 6.15*	74.61 ± 6.12*	61.81 ± 5.45*	14.94 ± 1.13*
**4d**	100 ± 9.93	91.42 ± 10.63	92.99 ± 9.77	91.48 ± 9.51	82.82 ± 8.68	70.04 ± 5.42*
**4e**	100 ± 9.93	91.42 ± 10.63	89.88 ± 9.92	82.11 ± 8.57	63.30 ± 7.13*	24.01 ± 3.45*
**4f**	100 ± 9.93	91.42 ± 10.63	92.36 ± 8.92	98.516 ± 9.21	97.58 ± 9.83	65.60 ± 6.92*
**4g**	100 ± 9.93	91.42 ± 10.63	72.28 ± 7.16*	65.24 ± 7.81*	66.03 ± 6.62*	46.79 ± 5.11*
**4h**	100 ± 9.93	91.42 ± 10.63	96.52 ± 9.56	71.77 ± 5.47*	68.17 ± 6.08*	58.56 ± 6.12*
**5a**	100 ± 9.93	91.42 ± 10.63	96.45 ± 9.89	91.64 ± 8.94	49.90 ± 5.12*	7.36 ± 0.09*
**5b**	100 ± 9.93	91.42 ± 10.63	89.53 ± 9.94	85.83 ± 8.84	65.27 ± 6.12*	12.16 ± 1.01*
Docetaxel	100 ± 9.93	91.42 ± 10.63	51.13 ± 7.41*	32.09 ± 4.82*	16.23 ± 4.16*	1.24 ± 0.75*

a The cell viability values obtained from amino acid derivatives of 7-chloroquinoline are shown as a percentage of absorbance (MTT) compared to the control. Each data point reflects the average of ten viability measurements. *p < 0.05.

**Table 4:** A2780 Cell Viability (%) Results with Compounds **4a**–**h** and **5a**,**b** ^a^

Compd	Control	DMSO	0.1 μg/mL	1 μg/mL	10 μg/mL	100 μg/mL
**4a**	100 ± 8.29	91.75 ± 9.84	84.86 ± 8.93	70.59 ± 6.12*	56.10 ± 5.22*	4.72 ± 0.53*
**4b**	100 ± 8.29	91.75 ± 9.84	87.84 ± 8.30	80.86 ± 8.85	15.31 ± 1.14*	4.82 ± 0.03*
**4c**	100 ± 8.29	91.75 ± 9.84	88.23 ± 8.91	70.71 ± 6.28*	55.14 ± 4.12*	54.87 ± 5.25*
**4g**	100 ± 8.29	91.75 ± 9.84	88.32 ± 8.91	81.41 ± 9.85	64.37 ± 7.22*	54.87 ± 6.13*
**4d**	100 ± 8.29	91.75 ± 9.84	94.57 ± 8.33	87.19 ± 9.68	70.38 ± 7.18*	64.83 ± 8.13*
**4e**	100 ± 8.29	91.75 ± 9.84	86.64 ± 10.79	85.15 ± 9.38	53.56 ± 6.21*	11.50 ± 1.11*
**4f**	100 ± 8.29	91.75 ± 9.84	96.49 ± 9.49	96.31 ± 9.85	92.20 ± 10.84	59.54 ± 6.31*
**4h**	100 ± 8.29	91.75 ± 9.84	92.47 ± 10.28	82.401 ± 9.83	62.76 ± 5.37*	63.03 ± 7.85*
**5a**	100 ± 8.29	91.75 ± 9.84	85.04 ± 9.19	65.56 ± 6.04*	34.53 ± 4.47*	5.19 ± 0.36*
**5b**	100 ± 8.29	91.75 ± 9.84	83.14 ± 11.43	70.17 ± 8.08*	66.84 ± 5.20*	6.02 ± 0.78*
Docetaxel	100 ± 8.29	90.43 ± 8.19	49.88 ± 7.01*	24.56 ± 4.96*	11.42 ± 3.10*	1.87 ± 0.54*

a The cell viability values obtained from amino acid derivatives of 7-chloroquinoline are shown as a percentage of absorbance (MTT) compared to the control. Each data point reflects the average of ten viability measurements. *p < 0.05.

**Table 5:** MCF-7 cell Viability (%) Results with Compounds **4a**–**h** and **5a**,**b** ^a^

Compd	Control	DMSO	0.1 μg/mL	1 μg/mL	10 μg/mL	100 μg/mL
**4a**	100 ± 9.32	94.78 ± 10.42	81.69 ± 9.95	68.93 ± 8.34*	56.43 ± 4.67*	5.39 ± 0.62*
**4b**	100 ± 9.32	94.78 ± 10.42	85.46 ± 8.97	79.75 ± 8.81	40.09 ± 6.44*	5.53 ± 0.34*
**4c**	100 ± 9.32	94.78 ± 10.42	61.09 ± 7.51*	60.73 ± 6.07*	65.35 ± 7.11*	58.06 ± 6.63*
**4d**	100 ± 9.32	94.78 ± 10.42	98.50 ± 11.93	92.05 ± 10.83	90.28 ± 8.96	66.88 ± 7.19*
**4e**	100 ± 9.32	94.78 ± 10.42	86.00 ± 9.58	64.52 ± 7.43*	52.74 ± 4.48*	31.6 ± 2.66*
**4f**	100 ± 9.32	94.78 ± 10.42	88.16 ± 10.82	88.63 ± 11.29	86.04 ± 8.37	55.32 ± 6.23*
**4g**	100 ± 9.32	94.78 ± 10.42	63.79 ± 6.55*	60.58 ± 5.12*	57.49 ± 6.03*	55.20 ± 5.21*
**4h**	100 ± 9.32	94.78 ± 10.42	90.20 ± 8.31	69.32 ± 5.28*	70.42 ± 8.71*	54.76 ± 4.27*
**5a**	100 ± 9.32	94.78 ± 10.42	76.58 ± 8.40	77.13 ± 9.33	63.21 ± 8.71*	6.72 ± 0.56*
**5b**	100 ± 9.32	94.78 ± 10.42	90.99 ± 8.34	80.54 ± 9.11	77.30 ± 7.89	15.73 ± 2.12*
Docetaxel	100 ± 9.32	94.78 ± 10.42	52.12 ± 8.44*	30.57 ± 5.78*	17.61 ± 4.73*	2.25 ± 0.71*

a The cell viability values obtained from amino acid derivatives of 7-chloroquinoline are shown as a percentage of absorbance (MTT) compared to the control. Each data point reflects the average of ten viability measurements. *p < 0.05.

The calculated log­(IC_50_) (μg/mL) dose response curves of **4a**–**h**, **5a**,**b**, and docetaxel for LNCaP cells is presented in Figure 1, the calculated log­(IC_50_) (μg/mL) dose response curve for A2780 cells is presented in Figure 2, and the calculated log­(IC_50_) (μg/mL) dose response curve for MCF-7 cells is presented in Figure 3.

**Figure 1:**
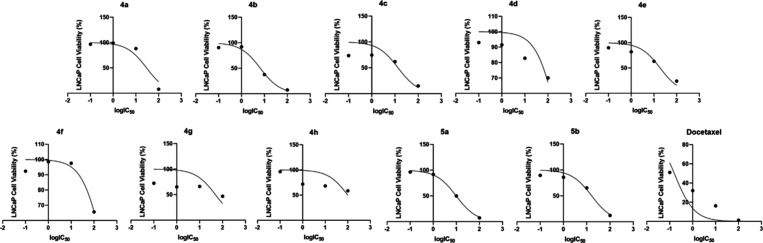
Calculated log­(IC_50_) (μg/mL) dose response curve of **4a**–**h**, **5a**,**b**, and docetaxel for LNCaP cells.

**Figure 2:**
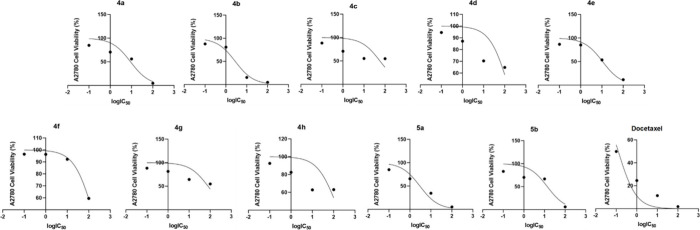
Calculated log­(IC_50_) (μg/mL) dose response curve of **4a**–**h**, **5a**,**b**, and docetaxel for A2780 cells.

**Figure 3:**
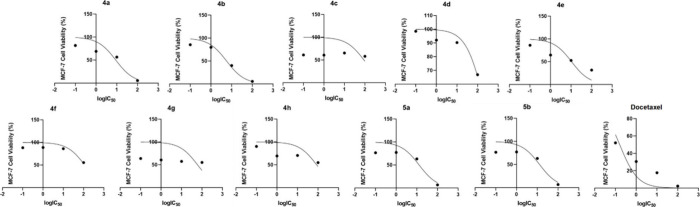
Calculated log­(IC_50_) (μg/mL) dose response curve of **4a**–**h**, **5a**,**b**, and docetaxel for MCF-7 cells.

#### 2.2.3 Molecular Docking

In order to explore the potential anticancer mechanism of the synthesized compounds, a molecular docking study was conducted on the most active derivatives, namely, compounds **4a**, **4b**, and **5a**, which exhibited IC_50_ values below 10 μg/mL on at least one cancer cell line. β-Tubulin was chosen as the molecular target because of its essential role in microtubule dynamics and its well-established involvement in the mechanism of action of docetaxel, a widely used anticancer agent. Docetaxel binds to the taxane site on polymerized β-tubulin, promoting microtubule stabilization, mitotic arrest in the G_2_/M phase, and subsequent apoptosis.^17^ Structural studies (PDB ID: 1TUB) have shown that docetaxel forms hydrogen bonds with Thr276, Gly360 and Arg278, as well as hydrophobic interactions with Pro360, Phe272, and Val23, among others, thereby contributing to the stabilization of microtubule architecture and the inhibition of cell division.^18^ By targeting the same binding site, this docking study aimed to evaluate whether derivatives **4a**, **4b**, and **5a** could exert their cytotoxic effects through a similar mechanism of β-tubulin stabilization.

The results, as shown in Figure 4, indicate that docetaxel binds to β-tubulin with a binding energy of −8.85 kcal/mol. Compounds **4a** and **4b** have values of −10.19 and −9.81 kcal/mol, respectively, indicating a higher theoretical affinity, while **5a** has a value of −8.74 kcal/mol, close to that of the native ligand. These data suggest that **4a** and **4b** could interact with β-tubulin in a more stable manner than docetaxel, while **5a** has a comparable affinity.

**Figure 4:**
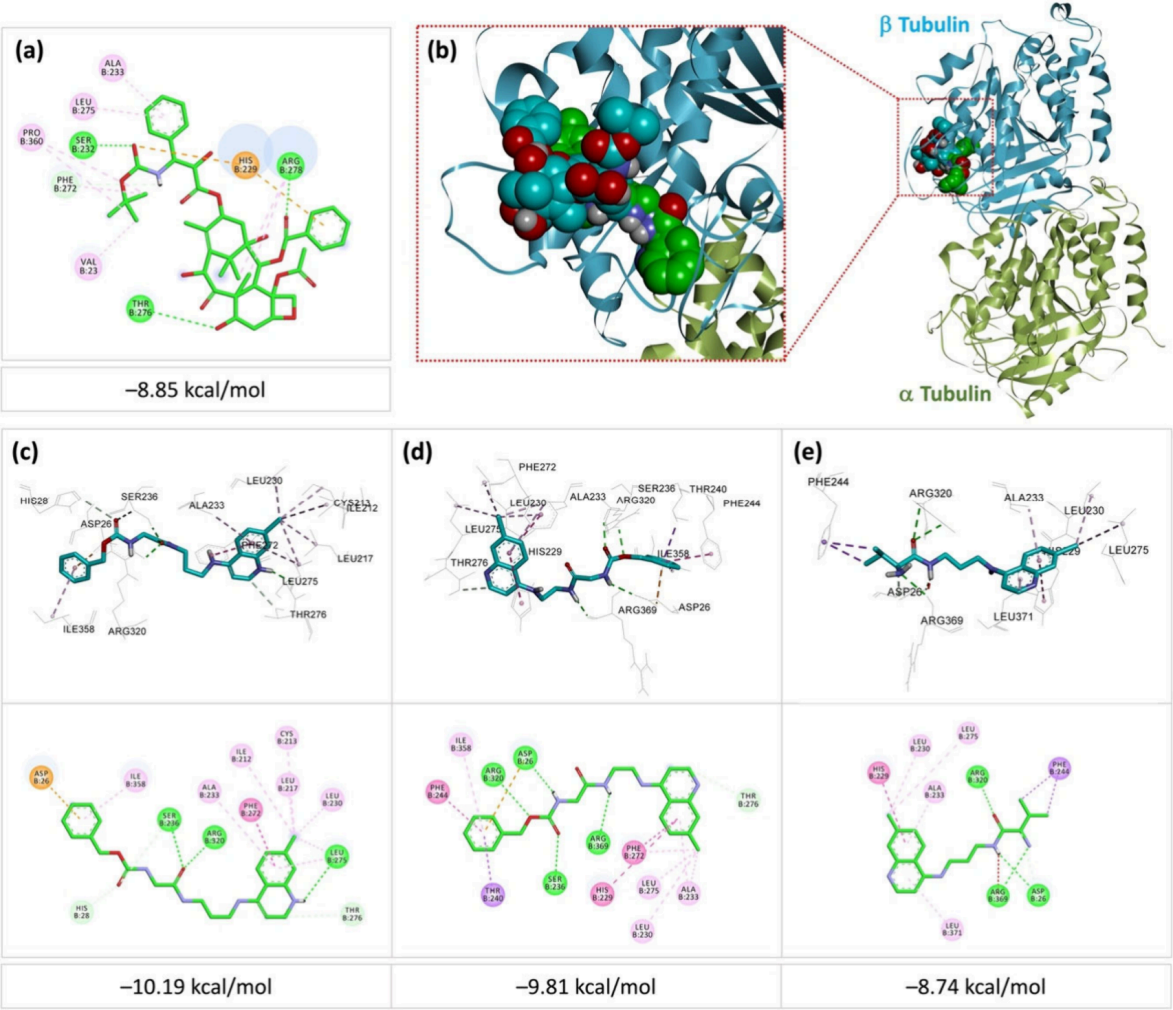
(a) 2D representation of the binding interactions of docetaxel with β-tubulin (1TUB); (b) superimposition of all docking poses of the studied compounds (green) and docetaxel (cyan) with β-tubulin (1TUB); (c, d, e) 3D orientations and 2D representations of the binding interactions of **4a**, **4b**, and **5a**, respectively, with β-tubulin (1TUB).

Analysis of interactions (Figure 4) reveals that docetaxel establishes a dense network of hydrogen bonds with key residues (Arg278, Ser232, and Thr276) and numerous hydrophobic interactions (Ala233, Leu275, Pro360, Phe272, and Val23), effectively stabilizing the molecule in the binding pocket. Compounds **4a** and **4b** partially reproduce these interactions, while forming additional contacts. **4a** establishes hydrophobic interactions with Leu275, Phe272, and Ala233, as well as two additional hydrogen bonds with Ser236 and Arg320, which are absent in the docetaxel−β-tubulin complex. **4b** forms four hydrogen bonds with Arg320, Asp26, Ser236, and Arg369, also absent in the docetaxel complex, and shares with the latter hydrophobic interactions with Leu275 and Ala233. **5a** adopts an interaction profile consisting of several favorable hydrogen-bond-type contacts with Arg320, Arg369, and Asp29, as well as hydrophobic interactions with Phe244, Leu371, His229, Ala233, Leu230, and Leu275. It shares with the docetaxel−β-tubulin complex hydrophobic interactions with Ala233, His229, and Leu275. Based on these results, it can be concluded that the compounds investigated have the potential to bind effectively to β-tubulin at the same site as docetaxel, thereby inhibiting cell division and promoting apoptosis. This mechanism could explain, at least in part, their observed cytotoxic effects.

## 3 Conclusion

New derivatives of 7-chloroquinoline, specifically carbamate and amino acid derivatives, have been synthesized, and their antioxidant and anticancer properties were evaluated. LNCaP (prostate lymph node carcinoma), A2780 (human ovarian), and MCF-7 (breast cancer) cell lines were used to assess the cytotoxic properties of novel 7-chloroquinolinyl benzyl amino carbamate derivatives. Compound **4b** demonstrated the best antitumor activity against LNCaP, A2780, and MCF-7 cell lines among all the compounds tested. Docking studies revealed that compounds **4a**, **4b**, and **5a** are potential inhibitors of microtubule depolymerization and may mimic the anticancer mechanism of docetaxel by stabilizing the microtubules.

## 4 Experimental Section

The chemicals and solvents used for the experiments were of synthetic grade. ^1^H NMR and ^13^C NMR data were obtained on Avance 400 and 101 MHz spectrometers in dimethyl sulfoxide (DMSO)-*d*_6_ and CDCl_3_. FTIR spectra were read on a PerkinElmer. TOF 6200 series/Q-TOF 6500 series (11.0.202.0) were used to obtain the mass of the compounds. A Gallenkamp brand melting point determination device was used to obtain the melting point of the compounds. All experiments were carried out at the Department of Chemistry, İnönü University, Malatya, Turkey. Precoated silica gel 60 F254 (mesh) were used to monitor the reactions via thin layer chromatography (TLC).

### 4.1 Synthesis of *N* ^1^-(7-Chloroquinolin-4-yl)­alkanediamine

A mixture of 4,7-dichloroquinoline (2.40g, 12 mmol) and alkanediamine (135 mmol) was stirred under microwave irradiation at 80 °C for 60 min. The reaction mixture was terminated by addition of dichloromethane and washed with 5% sodium hydroxide (2 × 50 mL). The organic portion was dried over anhydrous Na_2_SO_4_ and filtered, and the solvent was removed using a rotary evaporator. The crude solid was triturated with diethyl ether to afford **2a**,**b**.^19^

#### 4.1.1 Synthesis of *N* ^1^-(7-Chloroquinolin-4-yl)­ethan-1,2-diamine (**2a**)

Yield: 97.46%, mp 141–142 °C. ^1^H NMR (400 MHz, DMSO) δ 8.39 (d, *J* = 5.4 Hz, 1H, NH), 8.30 (d, *J* = 9.0 Hz, 1H, Ar), 7.78 (d, *J* = 1.8 Hz, 1H, Ar), 7.44 (d, *J* = 9.0 Hz, 1H, Ar), 7.38 (s, 1H, Ar), 6.50 (d, *J* = 5.4 Hz, 1H, Ar), 3.29 (br, 2H, NHC*H_2_* CH_2_NH-), 2.86 (t, *J* = 6.4 Hz, 2H, NHCH_2_C*H_2_* NH-). ^13^C NMR (101 MHz, DMSO) δ 152.4 (Ar- *C* _4_), 150.7 (Ar- *C* _2_), 149.5 (Ar- *C* _9_), 133.8 (Ar- *C* _7_), 127.9 (Ar- *C* _8_), 124.7­(Ar- *C* _5_), 124.5­(Ar- *C* _6_), 117.9 (Ar- *C* _10_), 99.2 (Ar- *C* _3_), 46.0 (C_11_), 40.3 (C_12_).

#### 4.1.2 Synthesis of *N* ^1^-(7-Chloroquinolin-4-yl)­propan-1,3-diamine (**2b**)

Yield: 95.20%, mp 143–144 °C. ^1^H NMR (400 MHz, DMSO) δ 8.39 (d, *J* = 5.2 Hz, 1H, NH), 8.26 (d, *J* = 9.0 Hz, 1H, Ar-H), 7.79 (s, 1H, Ar-H), 7.61 (s, 1H, Ar-H), 7.44 (d, *J* = 8.5 Hz, 1H, Ar-H), 6.46 (d, *J* = 5.3 Hz, 1H, Ar-H), 3.32 (t, *J* = 6.7 Hz, 2H, -NHC*H_2_* CH_2_CH_2_NH-), 2.71 (t, *J* = 6.3 Hz, 2H, -NHCH_2_CH_2_C*H_2_* NH-), 1.79–1.72 (m, 2H, -NHCH_2_C*H_2_* CH_2_NH-). ^13^C NMR (101 MHz, DMSO) δ 152.4 (Ar- *C* _4_), 150.6 (Ar- *C* _2_), 149.5 (Ar- *C* _9_), 133.8 (Ar- *C* _7_), 127.9 (Ar- *C* _8_), 124.5 (Ar- *C* _5_), 124.5 (Ar- *C* _6_), 117.9 (Ar- *C* _10_), 99.0 (Ar- *C* _3_), 41.0 (C_11_), 40.1 (C_12_), 31.1 (C_13_).

### 4.2 Synthesis of 7-Chloroquinolin-4-yl-aminocarbamate Derivatives (**4a**–**h**)

A mixture of *N* ^1^-(7-chloroquinolin-4-yl)­alkanediamine (2.5 mmol), Z-amino acid-Bt, and Boc-amino acid-Bt (5 mmol) was microwaved (100 W, 70 °C) in anhydrous THF for 1 h in the presence of 2.5 equiv of Et_3_N. After the reaction completed, a rotary evaporator was used to remove all volatile solvent. The solid was dissolved in DCM, and aqueous work up was carried out. The recrystallization of the crude product from a DCM/*n*-hexane mixture gave pure samples of **4a**–**h**.

#### 4.2.1 Benzyl (2-((3-((7-Chloroquinolin-4-yl)­amino)­propyl)­amino)-2-oxoethyl)­carbamate (**4a**)

Yield: 84%, mp 158–159 °C. IR (KBr) cm^–1^: 3300 (NH), 2952 (C–H aliphatic), 1687 (C=C), 1580, 1539 (C=O); 1246 (C=N), 808 (C–Cl). ^1^H NMR (400 MHz, DMSO) δ 8.40 (d, *J* = 5.3 Hz, 1H, Ar-H), 8.24 (t, *J* = 8.2 Hz, 1H, Ar-H), 8.01 (t, *J* = 5.4 Hz, 1H, N*H* of amide), 7.79 (d, *J* = 2.2 Hz, 1H, N*H*COO), 7.52 (t, *J* = 6.0 Hz, 1H, N*H*), 7.48–7.43 (m, 1H, Ar-H), 7.36 (d, *J* = 4.3 Hz, 4H, Ar-H), 7.31 (dd, *J* = 8.2, 3.9 Hz, 2H, Ar-H), 6.48 (d, *J* = 5.4 Hz, 1H, Ar-H), 5.04 (s, 2H, PhC*H_2_*), 3.61 (d, *J* = 6.0 Hz, 2H, COC*H_2_* NH-), 3.60–3.26 (m, 2H, NC*H*_2_CH_2_CH_2_NH-), 3.23–3.18 (m, 2H, NHCH_2_CH_2_C*H_2_* NH-), 1.81–1.73 (m, 2H, NCH_2_C*H_2_* CH_2_N-). ^13^C NMR (101 MHz, DMSO) δ 169.7, (NH*C*O), 157.0, (NH*C*OO), 152.40, 150.5, 149.5, 137.5, 133.9, 128.8, 128.3, 128.2, 128.0, 124.6, 124.5, 117.9, 99.2 (Ar-*C*), 66.0, 44.1, 41.0, 36.9, 28.3. HRMS *m*/*z* for C_22_H_23_ClN_4_O_3_ [M + H]^+^ calcd. 426.1459, found 427.1531 [M + H]^+^.

#### 4.2.2 Benzyl (2-((2-((7-Chloroquinolin-4-yl)­amino)­ethyl)­amino)-2-oxoethyl)­carbamate (**4b**)

Yield: 82.3%, mp 153–154 °C. IR (KBr) cm^–1^: 3307 (NH); 2927 (C–H-aliphatic); 1657 (C=C); 1581, 1529 (C=O); 1273 (C=N). ^1^H NMR (400 MHz, DMSO) δ 8.43 (d, *J* = 5.1 Hz, 1H, Ar-H), 8.21 (d, *J* = 8.4 Hz, 2H, Ar-H), 7.81 (d, *J* = 1.2 Hz, 1H, Ar-H), 7.54 (t, *J* = 8.0 Hz, 1H, N*H*), 7.46 (dd, *J* = 8.7, 1.2 Hz, 1H, Ar-H), 7.43–7.28 (m, 6H, 2NH + Ar-H), 6.58 (d, *J* = 5.2 Hz, 1H, Ar-H), 5.05 (s, 2H, PhC*H_2_*-), 3.65 (d, *J* = 5.5 Hz, 2H, COC*H_2_* NH-), 3.37 (br, 4H, -NHC*H*_2_C*H_2_* NH-). ^13^C NMR (101 MHz, DMSO) δ 170.3 (NH*C*O), 156.97 (NH*C*OO), 152.4, 150.5, 149.6, 137.5, 134.0, 128.8, 128.3, 128.2, 128.0, 124.6, 124.4, 117.9, 99.1 (Ar-C), 66.0, 44.2, 42.7, 37.7. HRMS *m*/*z* for C_21_H_21_ClN_4_O_3_ [M + H]^+^ calcd. 412.1302, found 413.1377 [M + H]^+^.

#### 4.2.3 Benzyl (*R*)-(1-((2-((7-Chloroquinolin-4-yl)­amino)­ethyl)­amino)-3-methyl-1-oxobutan-2-yl)­carbamate (**4c**)

Yield: 82.3%, mp 164–165 °C. IR (KBr) cm^–1^: 3390 (NH); 2976 (C–H-aliphatic); 1689 (C=C); 1585, 1532 (C=O); 1241 (C=N). ^1^H NMR (400 MHz, DMSO) δ 8.44 (d, *J* = 5.4 Hz, 1H, Ar-H), 8.41 (d, *J* = 5.3 Hz, 1H, NH), 8.20 (d, *J* = 9.0 Hz, 1H, Ar-H), 7.80 (d, *J* = 1.9 Hz, 1H, NH of amide), 7.49 (d, *J* = 8.8 Hz, 1H, N*H*COO), 7.44 (d, *J* = 7.2 Hz, 2H, Ar-H), 7.35 (d, *J* = 4.2 Hz, 4H, Ar-H), 7.31 (dd, *J* = 8.9, 4.3 Hz, 1H. Ar-H), 6.57 (d, *J* = 5.4 Hz, 1H, Ar-H), 5.13–4.85 (m, 2H, PhC*H_2_*), 3.80 (t, *J* = 7.8 Hz, 1H, NHC*H*CH­(C*H_3_*)_2_), 3.35 (d, *J* = 11.8 Hz, 4H, NC*H_2_* C*H_2_* NH-), 2.02–1.88 (m, 1H, NHCHC*H*(C*H_3_*)_2_), 0.81 (d, *J* = 6.7 Hz, 6H, NHCHCH­(C*H_3_*)_2_). ^13^C NMR (101 MHz, DMSO) δ 172.5, (NH*C*=O), 156.7, (NH*C*OO), 152.4, 150.5, 149.5, 137.5, 133.9, 128.8, 128.2, 128.1, 127.9, 124.6, 124.4, 117.9, 99.1, (Ar-*C*), 65.8, 61.1, 42.7, 37.6, 30.5, 19.7, 18.7. HRMS *m*/*z* for C_24_H_27_ClN_4_O_3_ [M + H]^+^ calcd. 454.1472, found 455.1843 [M + H]^+^.

#### 4.2.4 Benzyl (*S*)-(1-((2-((7-Chloroquinolin-4-yl)­amino)­ethyl)­amino)-4-(methylthio)-1-oxobutan-2-yl)­carbamate (**4d**)

Yield: 91%, mp 171–172 °C. IR (KBr) cm^–1^: 3401, 3291 (NH); 2928 (C–H-aliphatic); 1680 (C=C); 1581, 1532 (C=O); 1242 (C=N). ^1^H NMR (400 MHz, DMSO) δ 8.42 (d, *J* = 5.3 Hz, 1H, Ar-*H*), 8.26 (d, *J* = 5.3 Hz, 1H, N*H*), 8.19 (d, *J* = 9.0 Hz, 1H, Ar-*H*), 7.81 (d, *J* = 1.8 Hz, 1H, N*H* of amide), 7.60 (d, *J* = 7.9 Hz, 1H, Ar-*H*), 7.45 (d, *J* = 8.9 Hz, 1H, N*H*-COO), 7.40–7.24 (m, 6H, Ar-*H*), 6.58 (d, *J* = 5.4 Hz, 1H, Ar-H), 5.02 (q, *J* = 12.5 Hz, 2H, PhC*H_2_*), 4.15–4.00 (m, 1H, C*H*CH_2_CH_2_SCH_3_), 3.36 (d, *J* = 8.3 Hz, 4H, NHC*H_2_* C*H_2_* NH-), 2.44 (dd, *J* = 14.5, 7.9 Hz, 2H, CHCH_2_C*H_2_* SCH_3_),1.98 (s, 3H, CHCH_2_CH_2_SC*H_3_*), 1.88 (t, *J* = 10.1 Hz, 1H, CHC*H*_2_C*H^a^_2_* SCH_3_), 1.78 (dt, *J* = 19.1, 7.1 Hz, 1H, CHC*H*_2_C*H^b^_2_* SCH_3_). ^13^C NMR (101 MHz, DMSO) δ 172.7 (NH*C*O), 156.5 (NH*C*OO), 152.4, 150.5, 149.5, 137.4, 134.0, 128.8, 128.3, 128.2, 128.0, 124.6, 124.3, 117.9, 99.1, (Ar-C), 66.0, 54.5, 42.6, 37.8, 31.9, 30.2, 15.0, HRMS *m*/*z* for C_24_H_27_ClN_4_O_3_S [M + H]^+^ calcd. 486.1492, found 487.1566 [M + H]^+^.

#### 4.2.5 Benzyl (*S*)-(1-((3-((7-Chloroquinolin-4-yl)­amino)­propyl)­amino)-4-(methylthio)-1-oxobutan-2-yl)­carbamate (**4e**)

Yield: 88.2%, mp 156–157 °C. IR (KBr) cm^–1^: 3281 (NH); 2955 (C–H-aliphatic); 1689 (C=C); 1579, 1537 (C=O); 1237 (C=N). ^1^H NMR (400 MHz, DMSO) δ 8.40 (d, *J* = 5.3 Hz, 1H, Ar-*H*), 8.26 (d, *J* = 9.1 Hz, 1H, Ar-*H*), 8.08 (t, *J* = 5.4 Hz, 1H, N*H* of amide), 7.79 (d, *J* = 2.0 Hz, 1H, Ar-*H*), 7.55 (d, *J* = 7.9 Hz, 1H, N*H*COO), 7.46 (dd, *J* = 9.0, 1.9 Hz, 1H, Ar-H), 7.33 (dd, *J* = 20.8, 4.4 Hz, 6H, Ar-*H* + N*H*), 6.48 (d, *J* = 5.4 Hz, 1H), 5.09–4.98 (m, 2H, PhC*H_2_*), 4.07 (dd, *J* = 13.3, 8.5 Hz, 1H, NHC*H*CH_2_CH_2_SCH_3_), 3.28 (dt, *J* = 13.9, 6.8 Hz, 2H, NHC*H_2_* CH_2_CH_2_NH-), 3.21 (dd, *J* = 12.1, 6.0 Hz, 2H, NHCH_2_CH_2_C*H_2_* NH-), 2.49–2.40­(m, 2H, NHCHCH_2_C*H_2_* SCH_3_), 2.03 (s, 3H, NHCHCH_2_CH_2_SC*H_3_*), 1.93–1.74 (m, 4H, NHCH_2_C*H_2_* CH_2_NH- and NHCHC*H_2_* CH_2_SCH_3_). ^13^C NMR (101 MHz, DMSO) δ 172.1 (NH*C*O), 156.5 (NH*C*OO), 152.4, 150.5, 149.6, 137.5, 133.8, 128.8, 128.3, 128.2, 128.0, 124.5, 124.5, 118.0, 99.1 (Ar-C), 65.9, 54.5, 40.6, 36.9, 32.0, 30.2, 28.3, 15.1. HRMS *m*/*z* for C_25_H_29_ClN_4_O_3_S [M + H]^+^ calcd. 500.1649, found 501.1729 [M + H]^+^.

#### 4.2.6 *tert*-Butyl (*R*)-(1-((2-((7-Chloroquinolin-4-yl)­amino)­ethyl)­amino)-3-methyl-1-oxobutan-2-yl)­carbamate (**4f**)

Yield: 45%, mp 198–199 °C. IR (KBr) cm^–1^: 3408, 3382 (NH); 2945 (C–H-aliphatic); 1683 (C=C); 1582, 1530 (C=O); 1242 (C=N). ^1^H NMR (400 MHz, DMSO) δ 8.42 (d, *J* = 5.2 Hz, 1H, Ar-H), 8.16 (d, *J* = 8.5 Hz, 2H, Ar-H), 7.81 (br, 1H, NH of amide), 7.47 (d, *J* = 8.7 Hz, 1H, Ar-H), 7.34 (br, 1H, NH), 6.76 (d, *J* = 8.5 Hz, 1H, N*H*COO), 6.57 (d, *J* = 5.3 Hz, 1H, Ar-H), 3.75 (t, *J* = 7.5 Hz, 1H, NHC*H*CH­(CH_3_)_2_), 3.36 (br, 4H, NHC*H_2_* C*H_2_* NH-), 1.93 (d, *J* = 6.7 Hz, 1H, NHCHC*H*(CH_3_)_2_), 1.37 (s, 9H, COOC­(C*H_3_*)_3_), 0.82 (d, *J* = 4.5 Hz, 6H, NHCHCH­(C*H_3_*)_2_). ^13^C NMR (101 MHz, DMSO) δ 172.7, (NH*C*O), 156.0, (NH*C*OO), 152.5, 150.5, 149.5, 133.9, 128.0, 124.6, 124.3, 117.9, 99.1 (Ar-*C*), 78.5, 60.4, 42.8, 37.6, 30.6, 28.6, 19.7, 18.6. HRMS *m*/*z* for C_21_H_19_ClN_4_O_3_ [M + H]^+^ calcd. 420.1928, found 421.2051 [M + H]^+^.

#### 4.2.7 *tert*-Butyl (*R*)-(1-((3-((7-Chloroquinolin-4-yl)­amino)­propyl)­amino)-3-methyl-1-oxobutan-2-yl)­carbamate (**4g**)

Yield: 83.5%, mp 164–165 °C. IR (KBr) cm^–1^: 3393, 3349 (NH); 2986 (C–H-aliphatic); 1678 (C=C); 1589, 1514 (C=O); 1245 (C=N). ^1^H NMR (400 MHz, CDCl_3_) δ 8.40 (d, *J* = 5.0 Hz, 1H, Ar-*H*), 7.86 (d, *J* = 8.4 Hz, 2H, Ar-H), 7.30 (d, *J* = 8.8 Hz, 1H, N*H* of amide), 6.61 (br, 1H, -N*H*COO), 6.41 (br, 1H, NH), 6.29 (d, *J* = 5.0 Hz, 1H, Ar-H), 5.06 (s, 1H, Ar-H), 3.84 (t, *J* = 7.0 Hz, 1H, -COC*H*), 3.43–3.26 (m, 4H, NHC*H_2_* C*H_2_* C*H_2_* NH-), 2.11 (d, *J* = 5.7 Hz, 1H, C*H*(CH_3_)_2_), 1.75 (d, *J* = 3.7 Hz, 2H, NHCH_2_C*H_2_* CH_2_NH-), 1.37 (s, 9H, COOC­(C*H_3_*)_3_), 0.91 (dd, *J* = 13.2, 6.6 Hz, 6H, CH­(C*H_3_*)_2_). ^13^C NMR (101 MHz, CDCl_3_) δ 173.3 (NH*C*O), 156.1, (NH*C*OO), 151.7, 150.0, 149.1, 135.0, 128.3, 125.4, 122.0, 117.6, 98.5 (Ar-C), 80.3, 60.8, 39.0, 36.2, 30.4, 28.3, 28.2, 19.5, 18.0. HRMS *m*/*z* for C_22_H_31_ClN_4_O_3_ [M + H]^+^ calcd. 434.2035, found 435.2152 [M + H]^+^.

#### 4.2.8 *tert*-Butyl (*R*)-(1-((2-((7-Chloroquinolin-4-yl)­amino)­ethyl)­amino)-4-(methylthio)-1-oxobutan-2-yl)­carbamate (**4h**)

Yield: 79.4%, mp 187–188 °C. IR (KBr) cm^–1^: 3297 (NH); 2970 (C–H-aliphatic); 1682 (C=C); 1578, 1528 (C=O); 1273 (C=N). ^1^H NMR (400 MHz, DMSO) δ 9.48 (br, 1H, Ar-H), 8.61 (d, *J* = 9.1 Hz, 1H Ar-H), 8.55 (d, *J* = 6.9 Hz, 1H, NH), 8.22 (t, *J* = 5.0 Hz, 1H, NH of amide), 8.07 (s, 1H, Ar-H), 7.76 (d, *J* = 9.0 Hz, 1H, N*H*COO), 7.01 (d, *J* = 7.8 Hz, 1H, Ar-H), 6.93 (d, *J* = 7.0 Hz, 1H, Ar-H), 3.93 (d, *J* = 4.1 Hz, 1H, NHC*H*C*H*_2_C*H_2_* SCH_3_), 3.59 (d, *J* = 5.7 Hz, 2H, NHC*H_2_* CH_2_NH-), 2.38 (dd, *J* = 15.0, 8.4 Hz, 2H, NHCH_2_C*H_2_* NH-), 1.97 (s, 3H, NHCHCH_2_CH_2_SC*H_3_*), 1.80 (d, *J* = 6.6 Hz, 1H, NHCHC*H^a^*_2_C*H_2_* SCH_3_), 1.71 (dd, *J* = 14.1, 8.9 Hz, 1H, CHC*H^b^*_2_C*H_2_* SCH_3_), 1.34 (s, 9H, COOC­(C*H_3_*)_3_), 1.22 (d, *J* = 23.7 Hz, 1H, NHCHCH_2_C*H^a^*_2_SCH_3_), 0.86 (t, *J* = 6.4 Hz, 1H, NHCHCH_2_C*H^b^*_2_SCH_3_). ^13^C NMR (101 MHz, CDCl_3_) δ: 174.6 (NHCO), 150.9 (NHCOO), 150.5, 148.0, 135.4, 128.8, 127.5, 125.7, 122.2, 117.0, 98.2 (Ar-C), 80.6, 68.2, 53.8, 45.0, 38.6, 30.2, 28.2, 15.3. HRMS *m*/*z* for C_21_H_29_ClN_4_O_3_S [M + H]^+^ calcd. 452.1649, found 453.1723 [M + H]^+^.

### 4.3 General Procedure for the Synthesis of Unprotected 7-Chloroquinolin-4-yl-aminocarbamate Derivatives, **5a**,**b**

Boc-protected 7-chloroquinolin-4-yl-aminocarbamate derivatives (1.00 mmol) were dissolved in a 1:1 mixture of dichloromethane (DCM) and trifluoroacetic acid (TFA) (2 mL) and stirred for 1 h at room temperature. The reaction mixture was subjected to rotary evaporator, after which 20 mL of saturated sodium carbonate (Na_2_CO_3_) solution was added and stirred for 30 min, and extraction of the compound was done using ethyl acetate (EtOAc) (3 × 10 mL). The organic phase was dried by using anhydrous sodium sulfate (Na_2_SO_4_). The solvent was removed, and the pure compound was obtained by recrystallization from EtOAc/*n*-hexane.

#### 4.3.1 (*S*)-2-Amino-*N*-(3-((7-chloroquinolin-4-yl)­amino)­propyl)-3-methylbutanamide (**5a**)

Yield: 90%, mp 158–159 °C. IR (KBr) cm^–1^: 3275 (NH); 2964 (C–H-aliphatic); 1657 (C=C); 1582, (C=O). ^1^H NMR (400 MHz, DMSO) δ 8.45 (d, *J* = 5.4 Hz, 1H, Ar-H), 8.32 (d, *J* = 9.0 Hz, 1H, Ar-H), 8.03 (t, *J* = 5.2 Hz, 1H, NH of amide), 7.84 (d, *J* = 2.0 Hz, 1H, Ar-H), 7.51 (dd, *J* = 9.0, 2.0 Hz, 1H, Ar-H), 7.40 (t, *J* = 5.0 Hz, 1H, NH), 6.52 (d, *J* = 5.4 Hz, 1H, Ar-H), 3.27 (dd, *J* = 12.3, 6.0 Hz, 3H, NHC*H_2_* CH_2_CH_2_NH- + COC*H*NH_2_), 2.98 (d, *J* = 5.1 Hz, 1H, NHCHC*H*(CH_3_)_2_), 1.98–1.75 (m, 4H, NHCH_2_C*H_2_* C*H_2_* NH-), 0.92 (d, *J* = 6.8 Hz, 3H, NHCHCH­(C*H_3_*)_2_), 0.84 (d, *J* = 6.8 Hz, 3H, NHCHC*H*(C*H_3_*)_2_). ^13^C NMR (101 MHz, DMSO) δ 175.3 (NH*C*O), 152.4, 150.5, 149.6, 133.9, 128.0, 124.5, 124.5, 118.0, 99.1 (Ar-C) 60.7, 36.6, 32.1, 28.4, 20.1, 17.6. HRMS *m*/*z* for C_17_H_23_ClN_4_O_3_ [M + H]^+^ calcd. 334.1560, found 335.1637 [M + H]^+^.

#### 4.3.2 (*S*)-2-Amino-*N*-(2-((7-chloroquinolin-4-yl)­amino)­ethyl)-4-(methylthio)­butanamide (**5b**)

Yield: 90.3%, mp 154–155 °C. IR (KBr) cm^–1^: 3305 (NH); 2932 (C–H-aliphatic); 1650 (C=C); 1579, 1538 (C=O); 1223 (C=N). ^1^H NMR (400 MHz, DMSO) δ 8.42 (d, *J* = 5.4 Hz, 1H, Ar-H), 8.25 (d, *J* = 4.8 Hz, 1H, NH of amide), 8.19 (d, *J* = 9.0 Hz, 1H, Ar-H), 7.80 (d, *J* = 1.7 Hz, 1H, Ar-H), 7.47 (dd, *J* = 9.0, 1.7 Hz, 1H, Ar-H), 7.43 (d, *J* = 4.7 Hz, 1H, NH), 6.59 (d, *J* = 5.4 Hz, 1H, Ar-H), 3.37–3.33 (m, 4H, NHC*H_2_* C*H_2_* NH-), 3.24 (dd, *J* = 8.0, 4.7 Hz, 1H, COC*H*NH_2_), 2.48 (d, *J* = 7.2 Hz, 2H, NHCHCH_2_C*H_2_* SCH_3_), 1.98 (s, 3H, NHCHCH_2_CH_2_SC*H_3_*), 1.90–1.80 (m, 1H, NHCHC*H^a^_2_* CH_2_SCH_3_), 1.58 (td, *J* = 14.4, 7.5 Hz, 1H, NHCHC*H^a^_2_* CH_2_SCH_3_). ^13^C NMR (101 MHz, DMSO) δ 176.2 (NH*C*O), 152.4, 150.5, 149.5, 133.9, 128.0, 124.6, 124.3, 117.9, 99.1 (Ar-C) 54.5, 42.9, 37.6, 35.0, 30.3, 15.0. HRMS *m*/*z* for C_16_H_21_ClN_4_O_3_S [M + H]^+^ calcd. 352.1125, found 353.1204 [M + H]^+^.

### 4.4 In Vitro Antioxidant Studies

#### 4.4.1 DPPH Free Radical Inhibition Assay

Antioxidant activity was evaluated following the method in the literature^20^ with some modifications. 1,1-Diphenyl-2-picrylhydrazide (DPPH) was employed to assess the free radical scavenging activity. Solutions of the synthesized compounds were partitioned into five concentrations (12.5, 25.0, 37.5, 62.5, and 125 μg/mL) by using DMSO as the solvent. Each sample was incubated for 20 min at room temperature, and the absorbance was read at 517 nm using a UV spectrophotometer. The experiment was conducted in triplicate. Butylated hydroxytoluene (BHT) served as the standard control. The equation below was used to obtain the DPPH radical scavenging activity.

$$\mathrm{Scavenging effect}\left(\%\right)=\frac{\mathrm{Control absorbance}-\mathrm{Test absorbance}}{\mathrm{Control absorbance}}\times 100$$

#### 4.4.2 Human Cancer Cell Lines and Culture Conditions

Human cancer cell lines including LNCaP (Lymph Node Carcinoma of the Prostate), A2780 (ovarian carcinoma), and MCF-7 (breast cancer) were utilized for in vitro screening studies. These cell lines were maintained in RPMI-1640 medium supplemented with l-glutamine, 10% heat-inactivated fetal bovine serum, and 100 μg/mL penicillin–streptomycin. Cultures were incubated at 37 °C in a humidified atmosphere containing 5% CO_2_ (Panasonic, Japan).

#### 4.4.3 MTT Assay

The synthesized compounds (**4a**–**h**, **5a**,**b**) were evaluated for cytotoxic activity against the LNCaP, A2780, and MCF-7 cell lines using the MTT assay.^21^ This assay relies on the conversion of the yellow tetrazolium salt (MTT) into dark blue formazan crystals by metabolically active mitochondria, a process that can be quantified via a microplate reader. The MTT assay offers a nonradioactive method for assessing viable and proliferating cells.^22^ In brief, prostate, ovarian, and breast cancer cells were seeded at a density of 15 × 10^3^ cells/well in 96-well plates (final volume: 100 μL) and treated with varying concentrations of compounds **4a**–**h** and **5a**,**b** (0.1, 1, 10, and 100 μg/mL) in RPMI-1640. After 24 h incubation at 37 °C with 5% CO_2_, MTT solution (0.005 g/mL in phosphate-buffered saline) was added and incubated for an additional 3 h. The resulting formazan crystals were dissolved in 0.04 N isopropanol (100 mL), and absorbance was measured at 570 nm using a microplate reader (BioTek, Synergy HTX, USA).^21^ Control wells were used to calculate baseline absorbance, which was set as 100% cell viability. Absorbance values from solvent (DMSO-only) and compound-treated wells were compared to those from the control to determine percent cell viability. Each data point represented the mean of 10 replicates.^22^ All cellular outcomes were assessed relative to control cells as in our previous studies.^21–24^

#### 4.4.4 Molecular Docking

The molecular docking study was performed as follows. The structures of the tested molecules were initially sketched using MolView (https://molview.org) and then optimized using Avogadro with the Universal Force Field (UFF).^25^ The crystal structure of β-tubulin (PDB code: 1TUB) was retrieved from the Protein Data Bank.^26^ Protein preparation was carried out using Discovery Studio Visualizer by removing crystallographic water molecules and cocrystallized ligands. Polar hydrogens were added, and Gasteiger charges were assigned to the protein using AutoDockTools.^27^ The ligands were also prepared in AutoDockTools by adding polar hydrogens and assigning Gasteiger charges. Molecular docking was carried out using AutoDock Vina v1.2.7 ^28^ within the binding pocket of the native ligand, docetaxel. The docking search space was centered on the binding site of docetaxel. The docking protocol was validated by re-docking docetaxel into the active site of β-tubulin, and the predicted binding pose was compared with the crystallographic pose (Figure 5). A root-mean-square deviation (RMSD) of 2.46 Å was obtained. Although the RMSD value is higher than 2 Å, this is considered reasonable given the large size of the ligand and its high degree of conformational flexibility.

**Figure 5:**
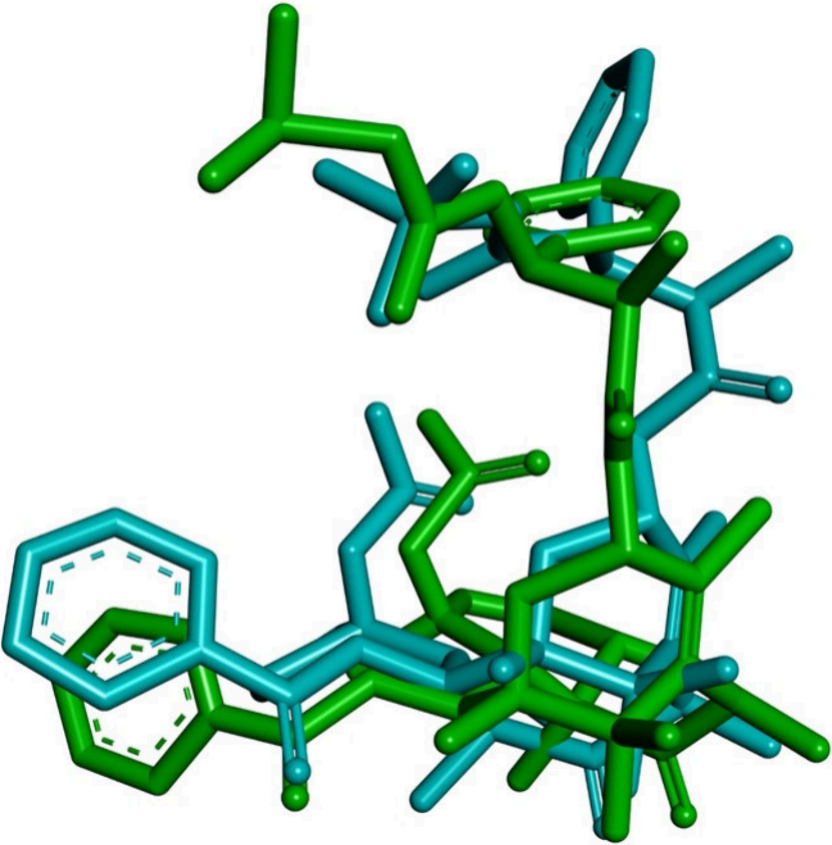
Re-docking validation of the native ligand docetaxel into β-tubulin (PDB ID: 1TUB). The experimental binding pose (green) is compared to the docking-predicted pose (cyan).

#### 4.4.5 Statistical Analysis

IBM SPSS Statistics 26.0 and MedCalc 11.0 for Windows were utilized for the analyses. Quantitative data were summarized using mean ± standard deviation (SD). The Shapiro–Wilk test was applied to assess the normality of distribution. The Kruskal–Wallis H test was employed to compare quantitative variables between groups, and when a significant difference was detected, pairwise comparisons were performed using the Bonferroni-corrected Mann–Whitney U test. A *p*-value of <0.05 was considered statistically significant. The IC_50_ values were calculated from % cell viability values obtained from the MTT assay using the GraphPad Prism 6 program.^29^

## Supplementary Material



## Data Availability

All data generated or analyzed during this study are included in the manuscript and Supporting Information.

## Notes

^1^H NMR, ^13^C NMR, HRMS, and FTIR spectra for all synthesized compounds (PDF)
