# Supplementary material for: Microwave Synthesis, Evaluation, and Docking Study of Amino Acid Derivatives of 7‑Chloroquinoline: Exploring Cytotoxic and Antioxidant Potentials
Source: ACS Omega. 2026 Jan 2;11(2):3184–94. doi: 10.1021/acsomega.5c09882 (PMC12824750; doi:10.1021/acsomega.5c09882)
Supplement: Supplementary file 1 [file ao5c09882_si_001.pdf]

Supplementary Material

Microwave synthesis, evaluation and docking study of amino acid derivatives of 7-chloroquinoline: Exploring cytotoxic, antioxidant potentials

James A. Ezugwu[1,6], Fatümetüzzehra Küçükbay[2], Samet Öz[3], Tuba Keskin[4], Houssem Boulebd[5], Suat Tekin[4], Hasan Küçükbay[6]*

[1]Department of Pure and Industrial Chemistry, University of Nigeria, Nsukka, 410001, Enugu State, Nigeria
[2]İnonu University, Faculty of Pharmacy, Department of Basic Pharmaceutical Sciences, 44280 Malatya, Turkey
[3] Osmaniye Korkut Ata University, Health Services Vocational School, Laboratory and Veterinary Health Department, Osmaniye Turkey
[4] İnönü University, Faculty of Medicine, Department of Physiology, 44280 Malatya, Turkey
[5] Laboratory of Synthesis of Molecules With Biological Interest, Department of Chemistry, Faculty of Exact Sciences, University Frères Mentouri Constantine 1, Constantine, Algeria
[6]İnönü University, Faculty of Arts and Sciences, Department of Chemistry, 44280 Malatya, Turkey

*Email: hasan.kucukbay@inonu.edu.tr

S1. Synthesis and Characterisation

S1.1: Spectra data of *N*[1]-(7-chloroquinolin-4-yl)ethane-1,2-diamine (2a)

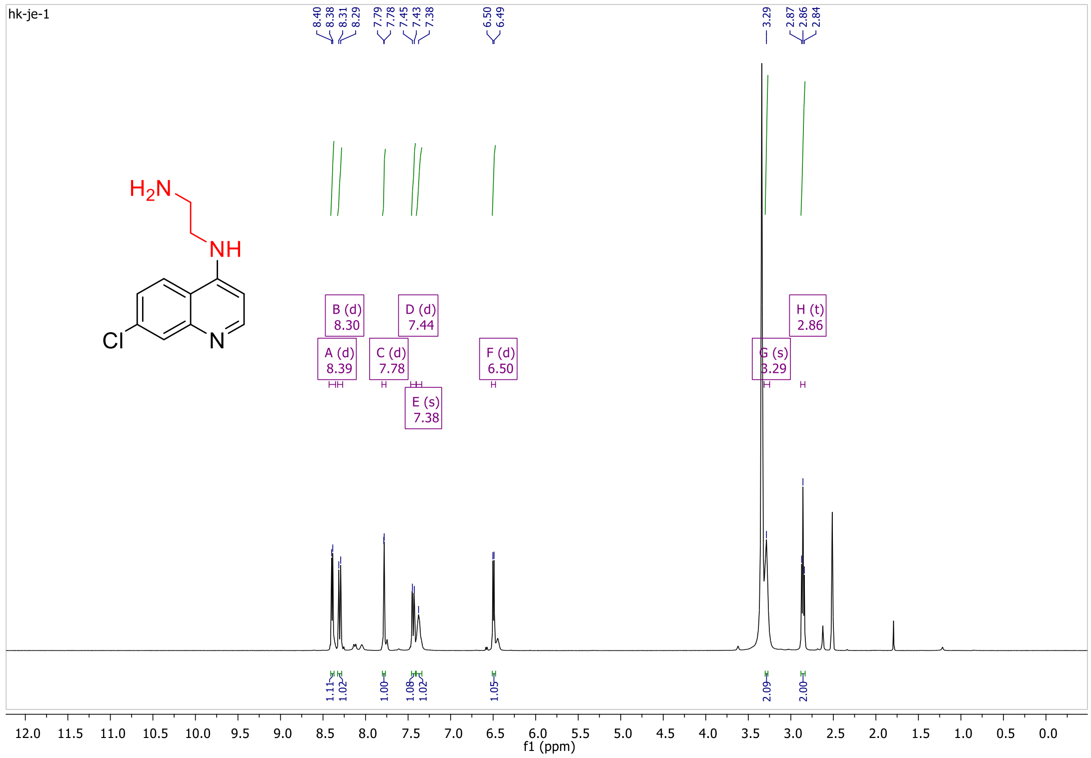

Figure S1: ^{1}H NMR Spectrum of *N*[1]-(7-chloroquinolin-4-yl)ethane-1,2-diamine (**2a**)

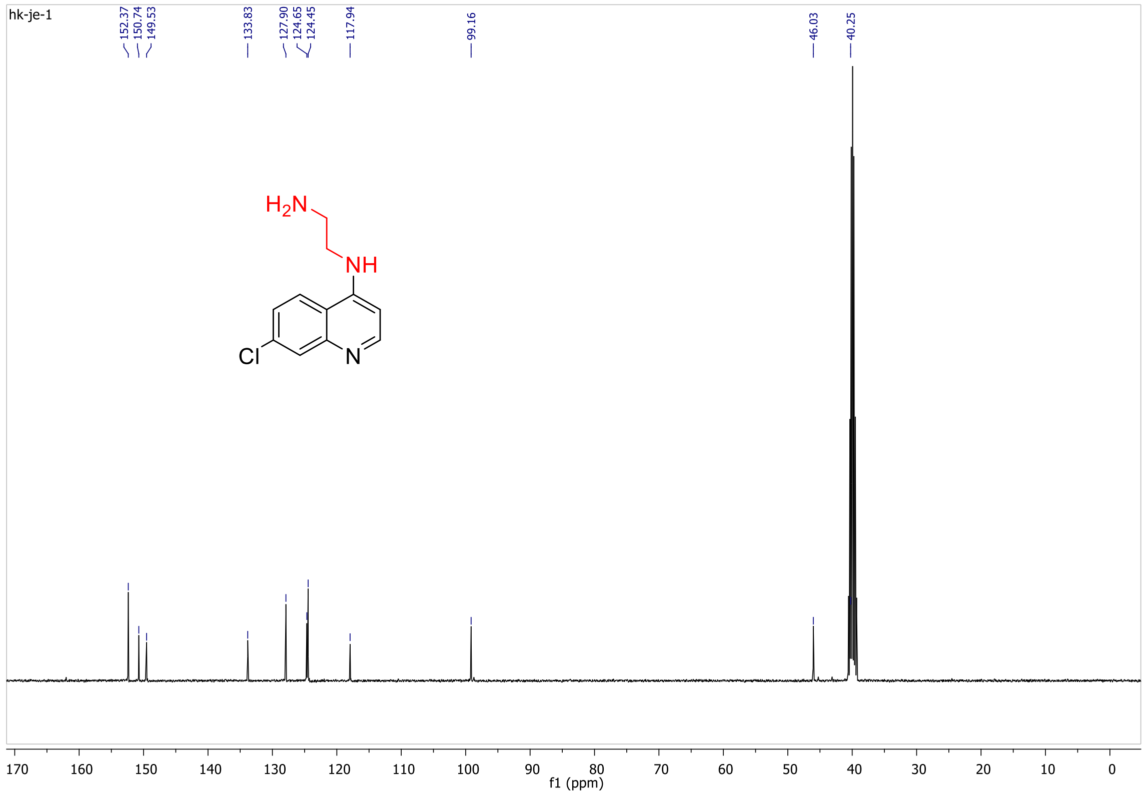

Figure S2: ^{13}C NMR Spectrum of *N*[1]-(7-chloroquinolin-4-yl)ethane-1,2- diamine (**2a**)

S1.2: Spectra data of N^1-(7-chloroquinolin-4-yl)propane-1,3-diamine (2b)

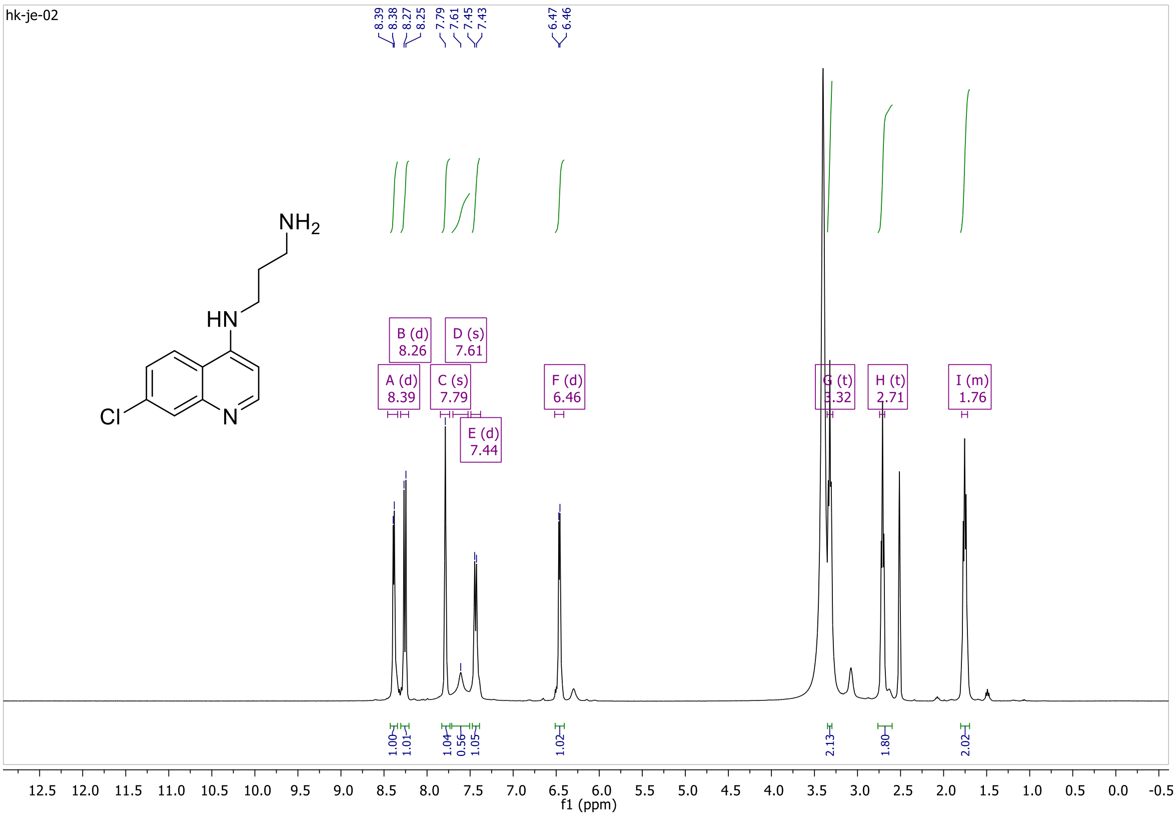

Figure S3: ^{1}H NMR Spectrum of N^1-(7-chloroquinolin-4-yl)propane-1,3-diamine (**2b**)

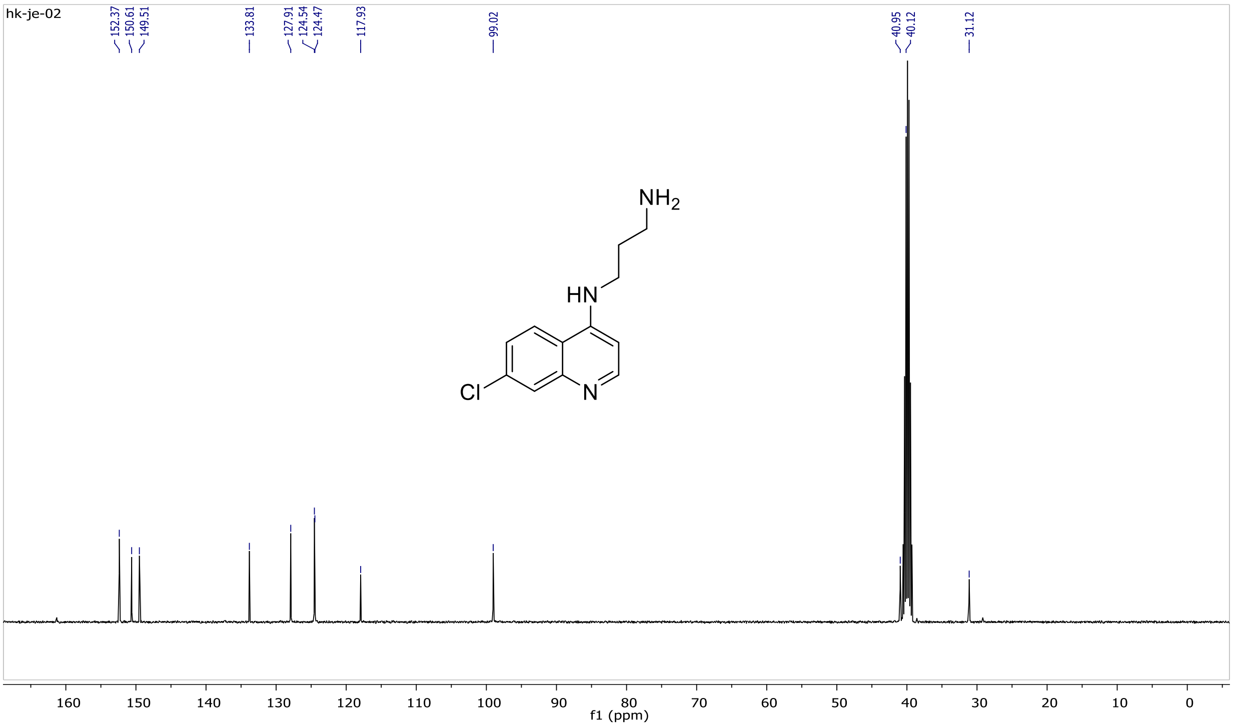

Figure S4: ^{13}C NMR Spectrum of N^1-(7-chloroquinolin-4-yl propane-1,2-diamine (**2b**)

S1.3: Spectra data of Benzyl (2-((3-((7-chloroquinolin-4-yl)amino)propyl)amino)-2-oxoethyl)carbamate (**4a**)

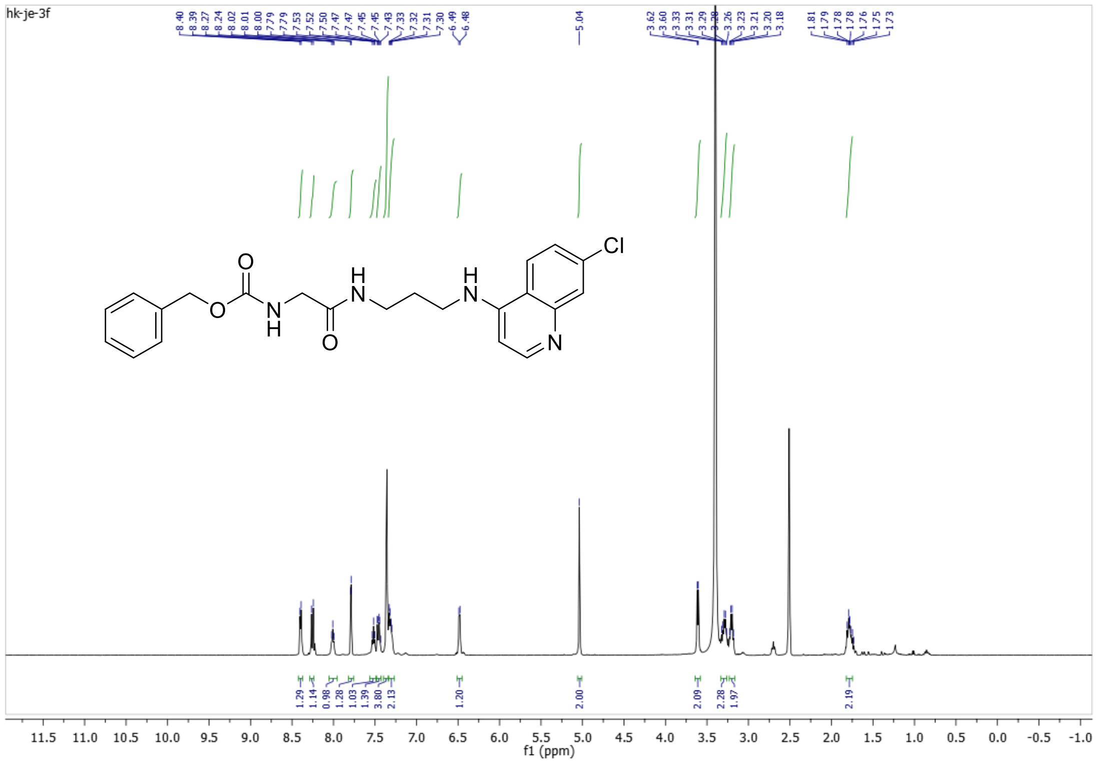

Figure S5: ^{1}H NMR Spectra of Benzyl (2-((3-((7-chloroquinolin-4-yl)amino)propyl)amino)-2-oxoethyl)carbamate (**4a**)

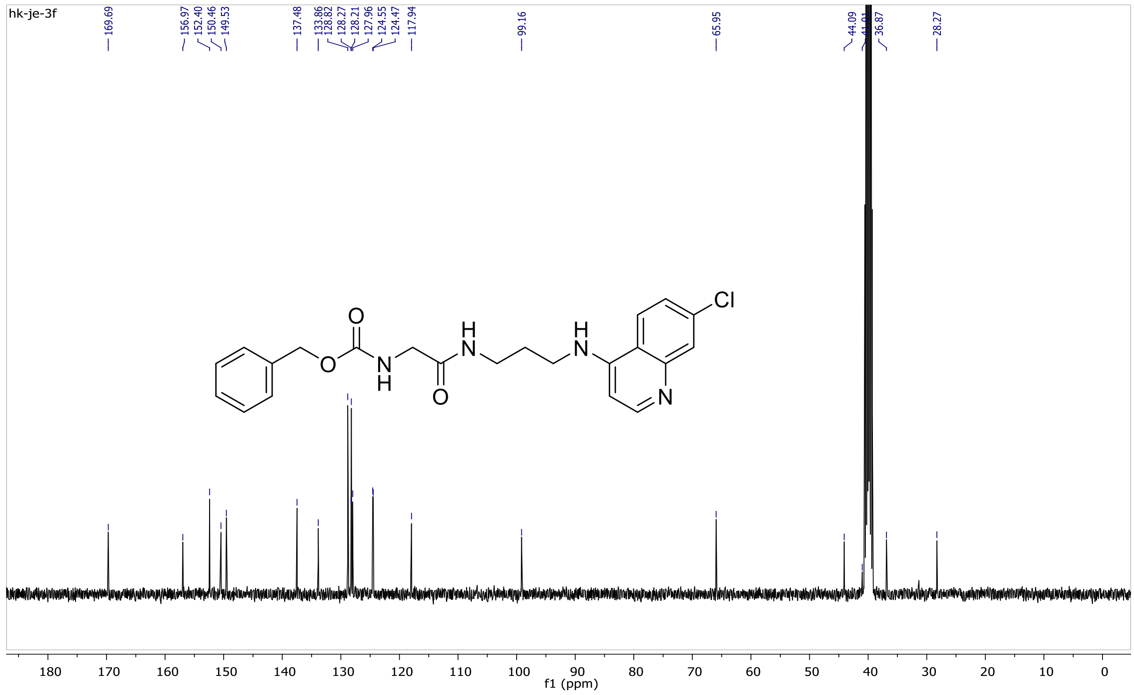

Figure S6: ^{13}C NMR Spectra of Benzyl (2-((3-((7-chloroquinolin-4-yl)amino)propyl)amino)-2-oxoethyl)carbamate (**4a**)

Qualitative Analysis Report

**Data Filename**	HK18.d	**Sample Name**	HK18
**Sample Type**	Sample	**Position**	P1-B9
**Instrument Name**	6530B LC Q-TOF	**User Name**	OQADMIN (oqadmin)
**Acq Method**	ESI_Pos.m	**Acquired Time**	3/14/2025 3:50:45 PM (UTC+03:00)
**IRM Calibration Status**	Success	**DA Method**	hcan.m
**Comment**			

**Sample Group**		**Info.**	
**Stream Name**	LC 1	**Method Version**	2025-0312-1046-26927
**Override DA Method Version**		**Data File Version**	2025-0314-1250-42243
**Acquisition Workstation**	DESKTOP-L73MD3C	**DA Workstation**	DESKTOP-L73MD3C
**Acquisition Time (Local)**	3/14/2025 3:50:45 PM (UTC+03:00)	**Acquisition SW Version**	6200 series TOF/6500 series Q-TOF (11.0.203.0)
**QTOF Driver Version**	11.00.00	**QTOF Firmware Version**	15.851
**Tune Mass Range Max.**	3200		

Chromatograms

Fragmentor Voltage 90 **Collision Energy** 0 **Ionization Mode** ESI

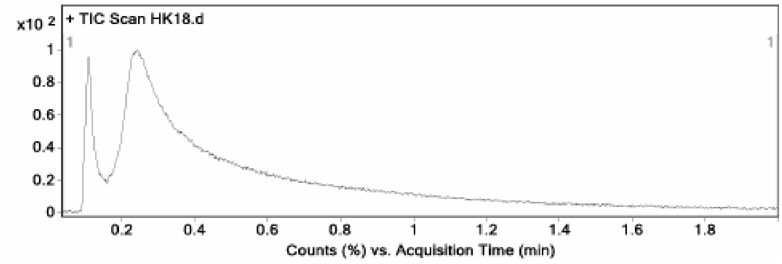

Spectra

Fragmentor Voltage	Collision Energy	Ionization Mode
90	0	ESI

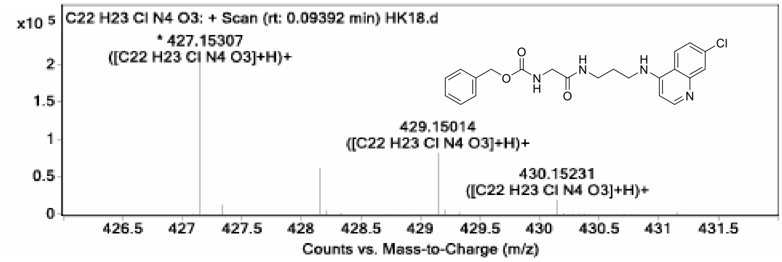

Peak List

Figure S7: HRMS of Benzyl (2-((3-((7-chloroquinolin-4-yl)amino)propyl)amino)-2-oxoethyl)carbamate (**4a**)

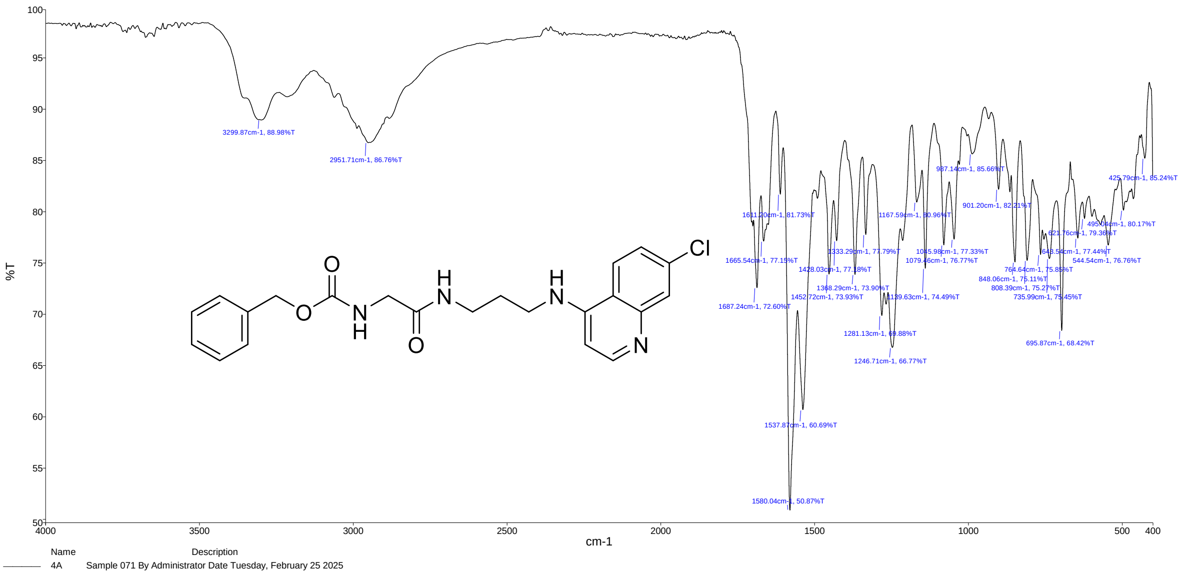

Figure S8: FTIR of Benzyl (2-((3-((7-chloroquinolin-4-yl)amino)propyl)amino)-2-oxoethyl)carbamate (**4a**)

S1.4: Spectra data of benzyl (2-((2-((7-chloroquinolin-4-yl)amino)ethyl)amino)-2-oxoethyl)carbamate (**4b**)

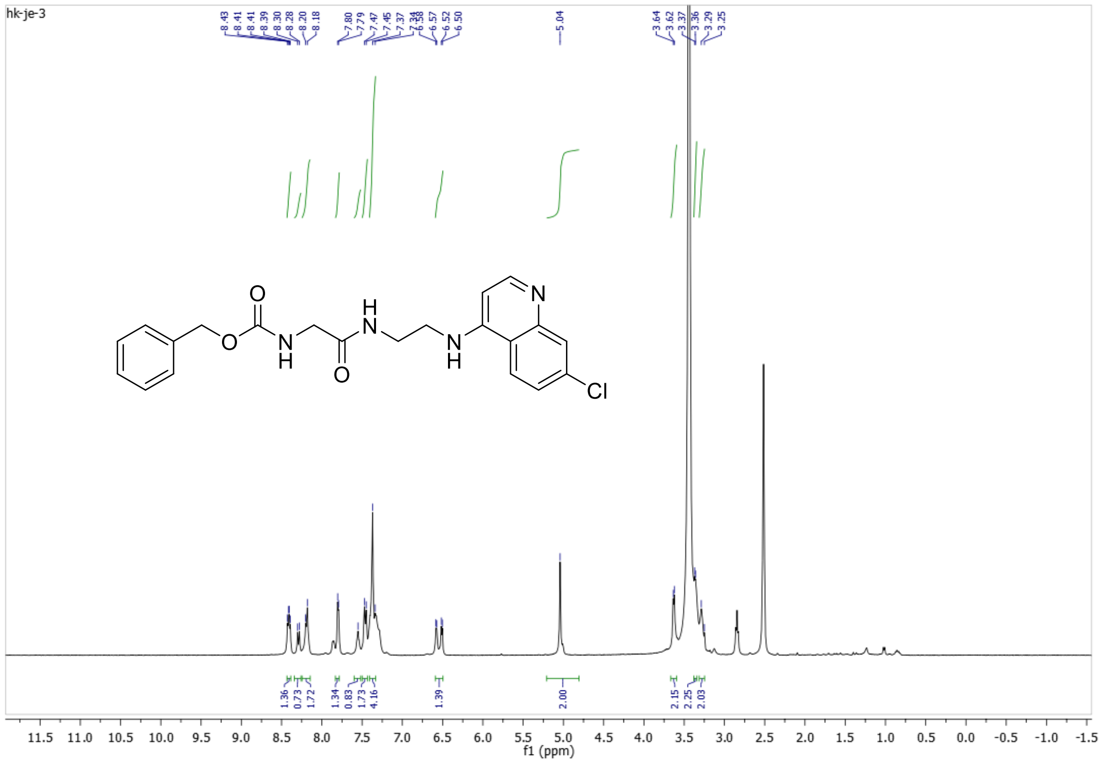

Figure S9: ¹H NMR Spectrum of benzyl (2-((2-((7-chloroquinolin-4-yl)amino)ethyl)amino)-2-oxoethyl)carbamate (**4b**)

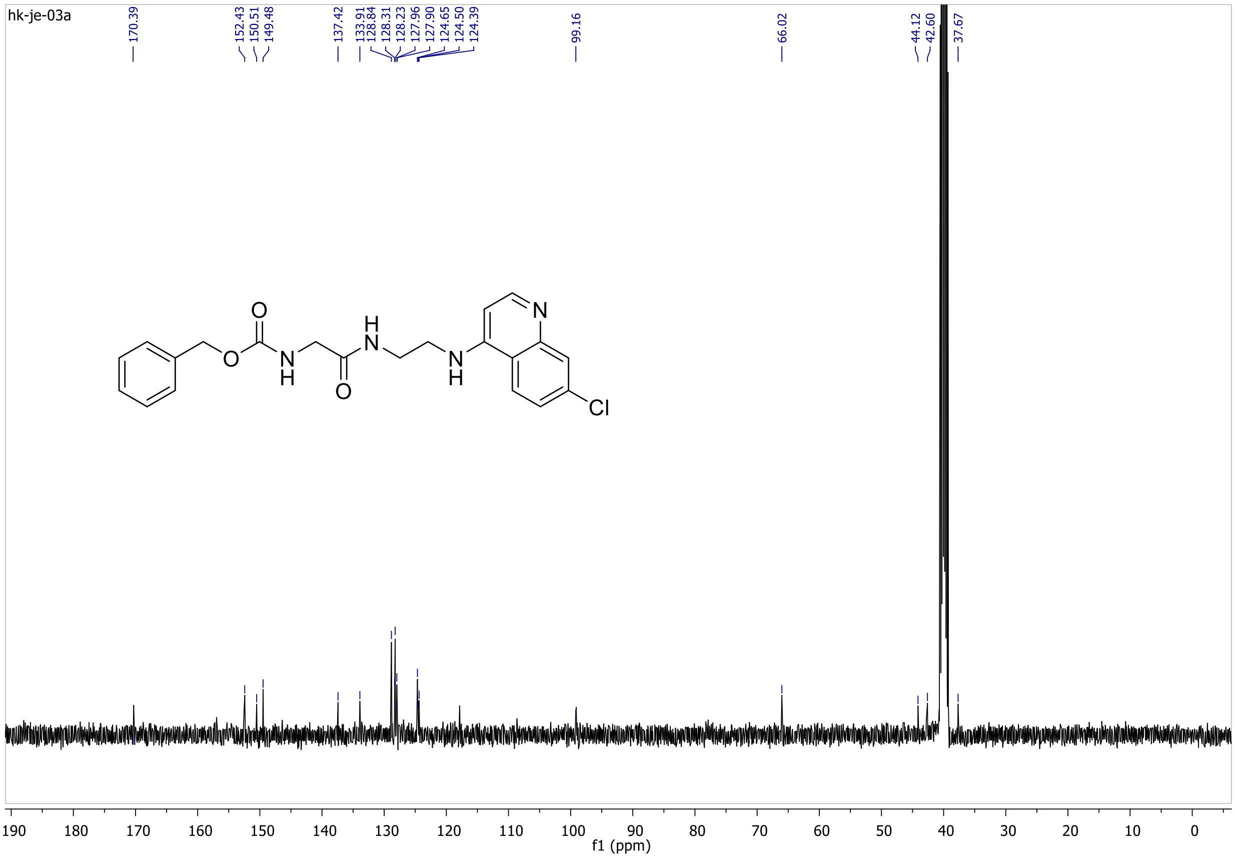

Figure S10: ^{13}C NMR Spectrum of benzyl (2-((2-((7-chloroquinolin-4-yl)amino)ethyl)amino)-2-oxoethyl)carbamate (**4b**)

Qualitative Analysis Report

**Data Filename**	HK21.d	**Sample Name**	HK21
**Sample Type**	Sample	**Position**	P1-C3
**Instrument Name**	6530B LC Q-TOF	**User Name**	OQADMIN (oqadmin)
**Acq Method**	ESI_Pos.m	**Acquired Time**	5/6/2025 5:19:46 PM (UTC+03:00)
**IRM Calibration Status**	Success	**DA Method**	hcan.m
**Comment**			

**Sample Group**		**Info.**	
**Stream Name**	LC 1	**Method Version**	2025-0505-0809-01740
**Override DA Method Version**		**Data File Version**	2025-0506-1419-41108
**Acquisition Workstation**	DESKTOP-L73MD3C	**DA Workstation**	DESKTOP-L73MD3C
**Acquisition Time (Local)**	5/6/2025 5:19:46 PM (UTC+03:00)	**Acquisition SW Version**	6200 series TOF/6500 series Q-TOF (11.0.203.0)
**QTOF Driver Version**	11.00.00	**QTOF Firmware Version**	15.851
**Tune Mass Range Max.**	3200		

Chromatograms

Fragmentor Voltage 90 **Collision Energy** 0 **Ionization Mode** ESI

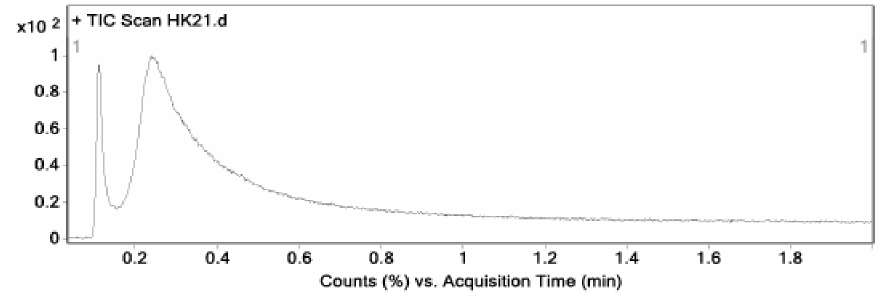

Spectra

Fragmentor Voltage	Collision Energy	Ionization Mode
90	0	ESI

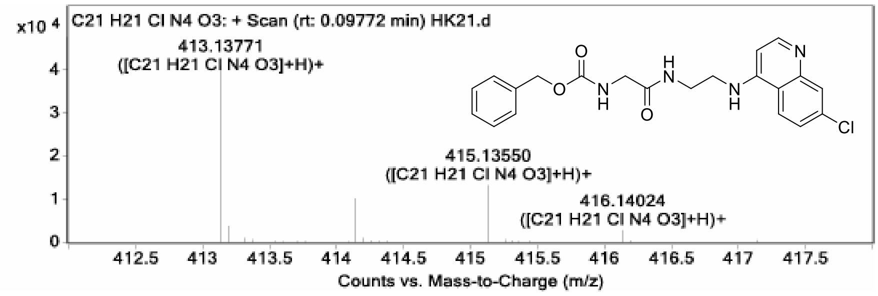

Peak List

Figure S11: HRMS of Benzyl (2-((2-((7-chloroquinolin-4-yl)amino)ethyl)amino)-2-oxoethyl)carbamate (**4b**)

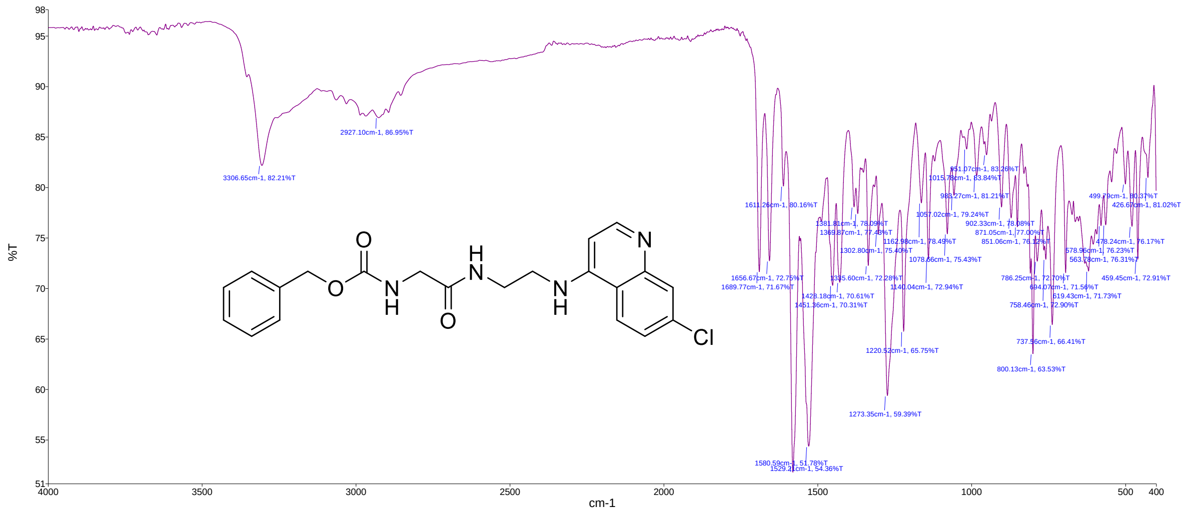

Figure S12: FTIR of Benzyl (2-((2-((7-chloroquinolin-4-yl)amino)ethyl)amino)-2-oxoethyl)carbamate (**4b**)

S1.5: Spectra data of Benzyl (1-((2-((7-chloroquinolin-4-yl)amino)ethyl)amino)-3-methyl-1-oxobutan-2-yl)carbamate (**4c**).

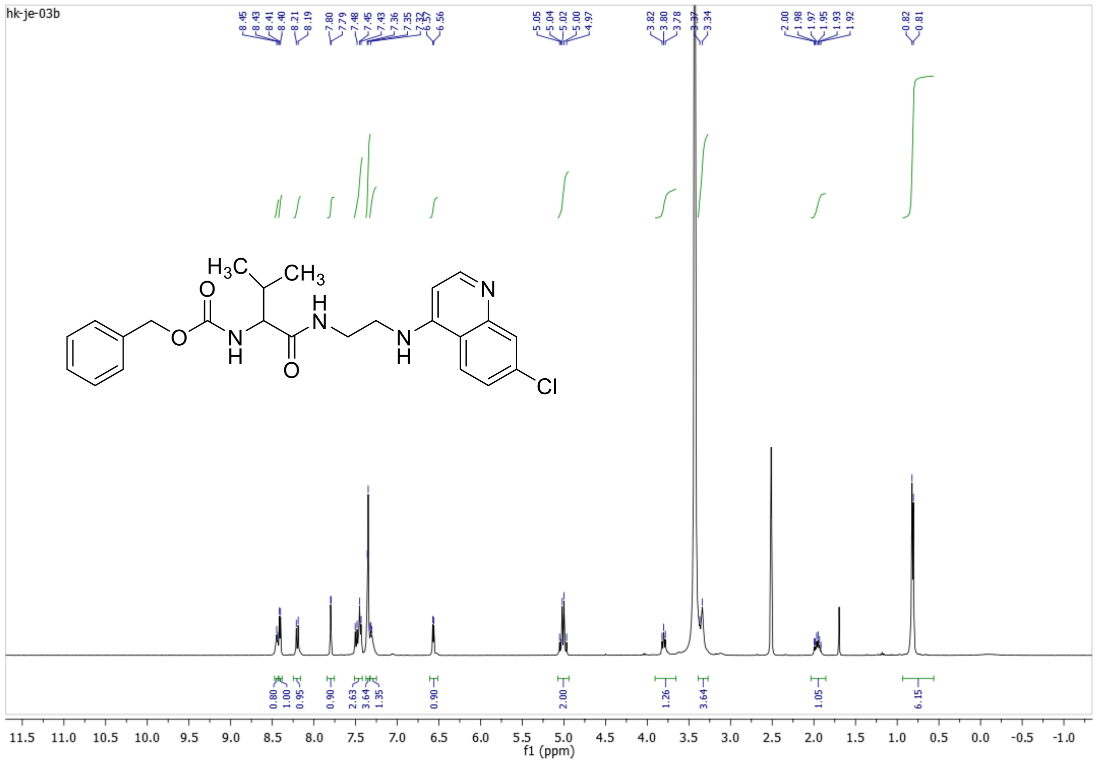

Figure S13: ^{1}H NMR Spectra of Benzyl (1-((2-((7-chloroquinolin-4-yl)amino)ethyl)amino)-3-methyl-1-oxobutan-2-yl)carbamate (**4c**).

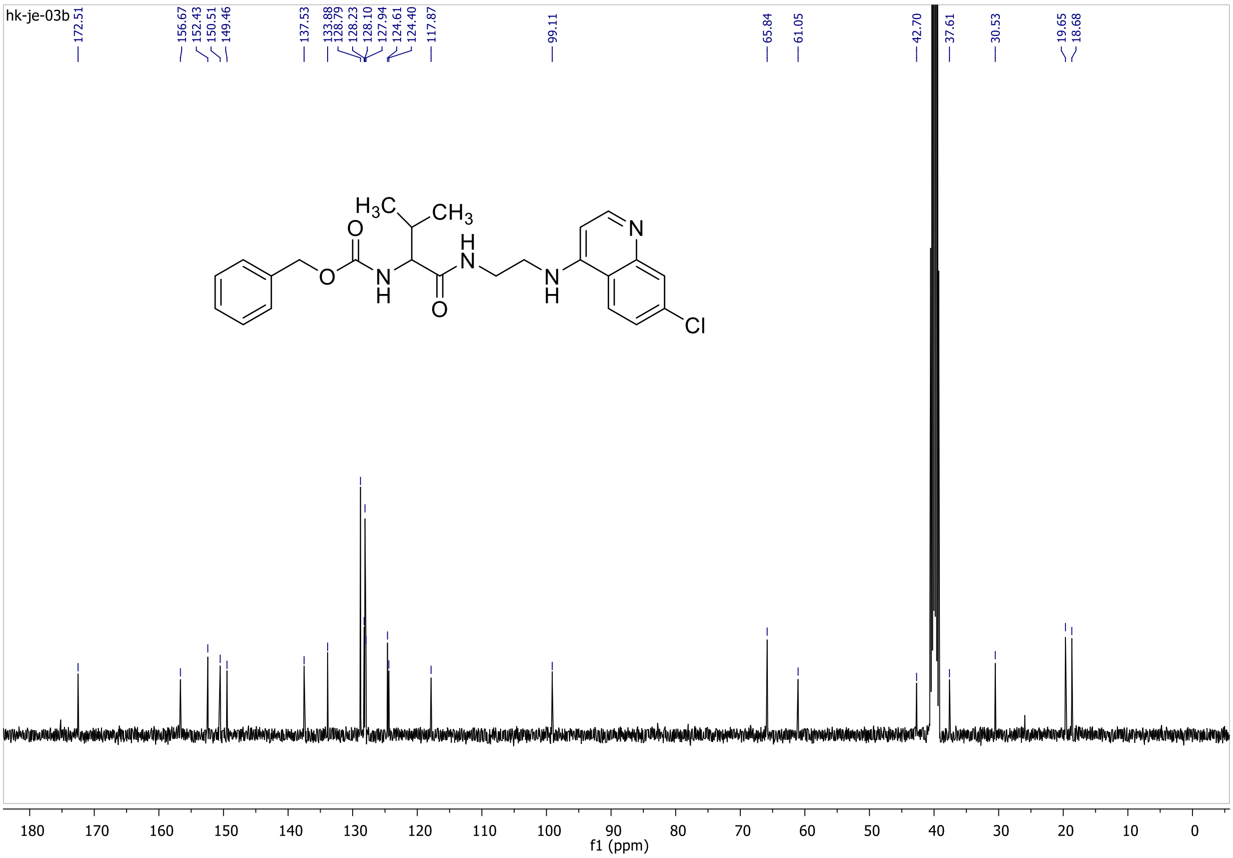

Figure S14: ^{13}C NMR Spectra of Benzyl (1-((2-((7-chloroquinolin-4-yl)amino)ethyl)amino)-3-methyl-1-oxobutan-2-yl)carbamate (**4c**).

Qualitative Analysis Report

**Data Filename**	HK16.d	**Sample Name**	HK16
**Sample Type**	Sample	**Position**	P1-B7
**Instrument Name**	6530B LC Q-TOF	**User Name**	OQADMIN (oqadmin)
**Acq Method**	ESI_Pos.m	**Acquired Time**	3/14/2025 3:45:18 PM (UTC+03:00)
**IRM Calibration Status**	Success	**DA Method**	hcan.m
**Comment**			

**Sample Group**		**Info.**	
**Stream Name**	LC 1	**Method Version**	2025-0312-1046-26927
**Override DA Method Version**		**Data File Version**	2025-0314-1245-14975
**Acquisition Workstation**	DESKTOP-L73MD3C	**DA Workstation**	DESKTOP-L73MD3C
**Acquisition Time (Local)**	3/14/2025 3:45:18 PM (UTC+03:00)	**Acquisition SW Version**	6200 series TOF/6500 series Q-TOF (11.0.203.0)
**QTOF Driver Version**	11.00.00	**QTOF Firmware Version**	15.851
**Tune Mass Range Max.**	3200		

Chromatograms

Fragmentor Voltage 90 **Collision Energy** 0 **Ionization Mode** ESI

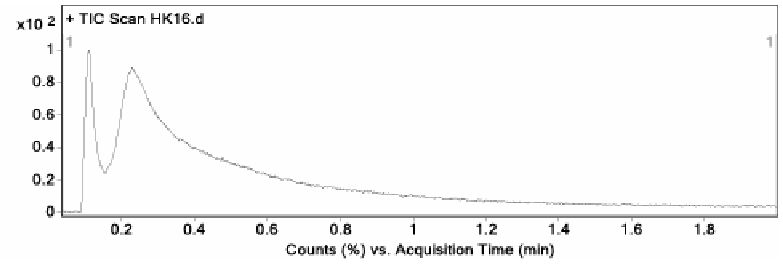

Spectra

**Fragmentor Voltage**	**Collision Energy**	**Ionization Mode**
90	0	ESI

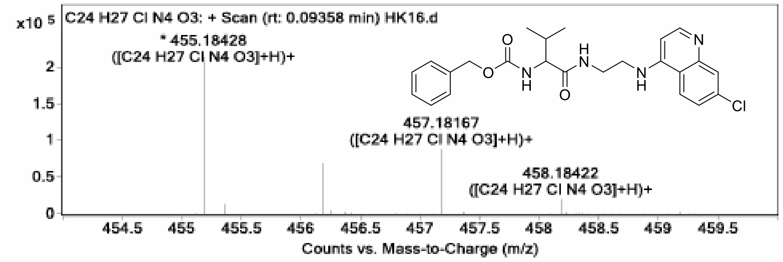

Figure S15: HRMS of Benzyl (1-((2-((7-chloroquinolin-4-yl)amino)ethyl)amino)-3-methyl-1-oxobutan-2-yl)carbamate (**4c**).

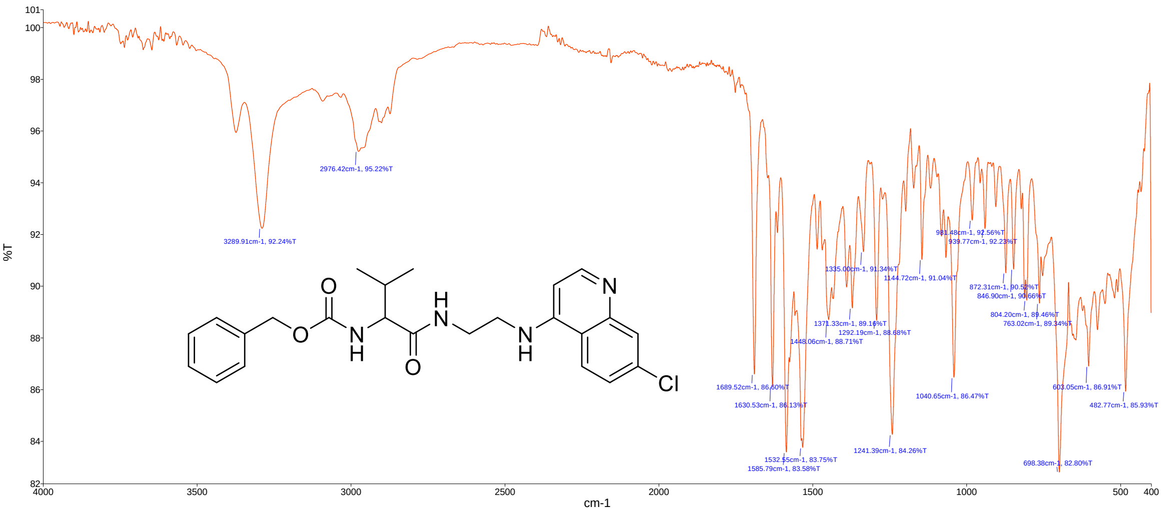

Figure S16: FTIR of Benzyl (1-((2-((7-chloroquinolin-4-yl)amino)ethyl)amino)-3-methyl-1-oxobutan-2-yl)carbamate (**4c**).

S1.6: Spectra data of Benzyl (1-((2-((7-chloroquinolin-4-yl)amino)ethyl)amino)-4-(methylthio)-1-oxobutan-2-yl)carbamate (**4d**)

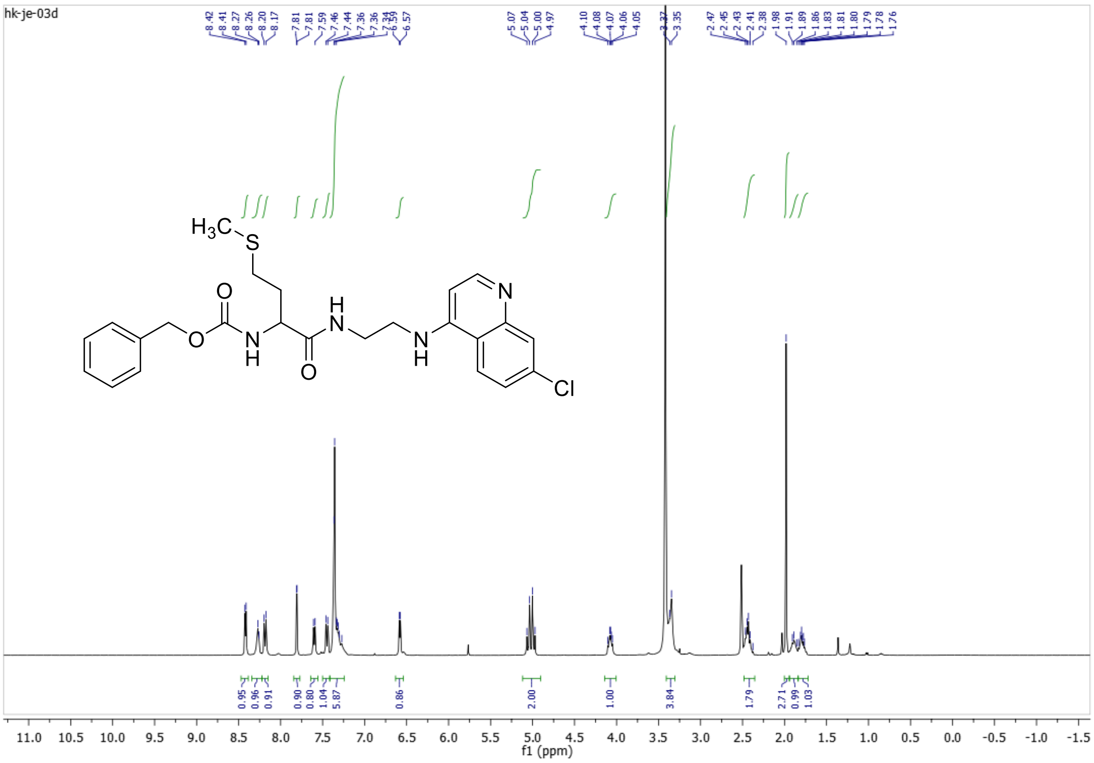

Figure S17: ^{1}H NMR Spectra of Benzyl (1-((2-((7-chloroquinolin-4-yl)amino)ethyl)amino)-4-(methylthio)-1-oxobutan-2-yl)carbamate (**4d**)

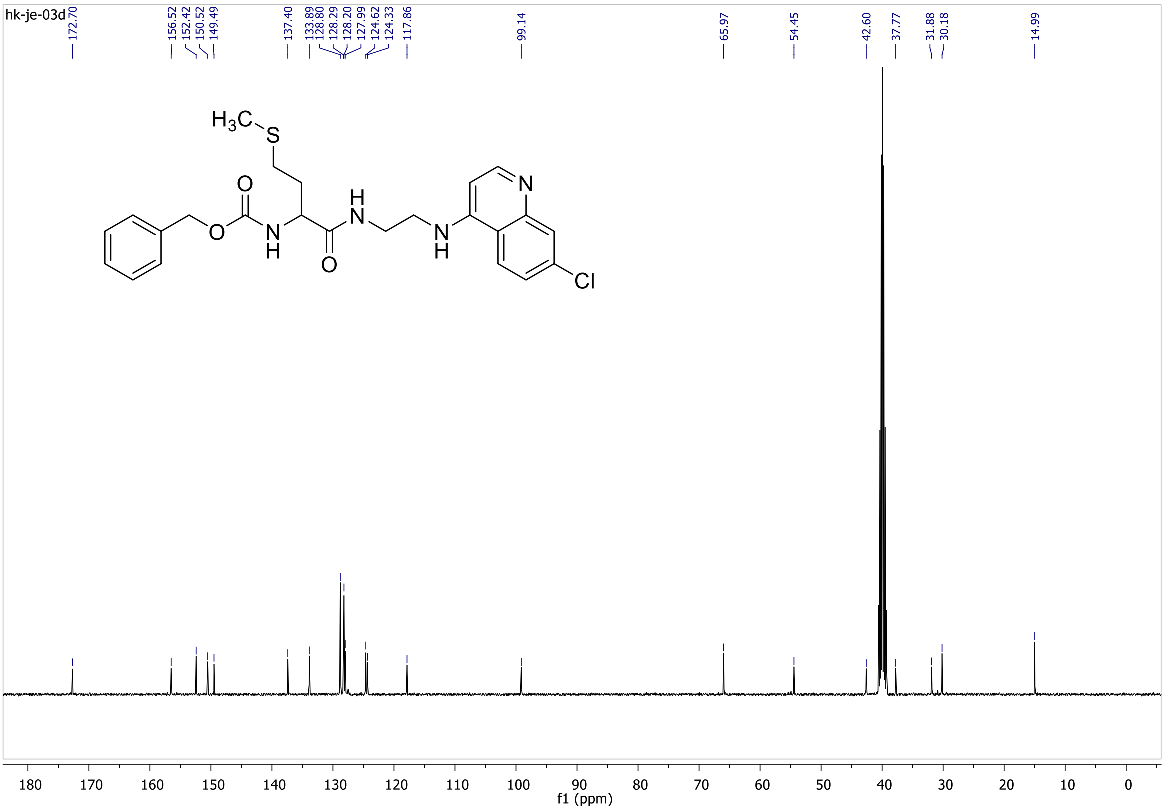

Figure S18: ^{13}C NMR Spectra of Benzyl (1-((2-((7-chloroquinolin-4-yl)amino)ethyl)amino)-4-(methylthio)-1-oxobutan-2-yl)carbamate (**4d**)

Qualitative Analysis Report

**Data Filename**	HK17.d	**Sample Name**	HK17
**Sample Type**	Sample	**Position**	P1-B8
**Instrument Name**	6530B LC Q-TOF	**User Name**	OQADMIN (oqadmin)
**Acq Method**	ESI_Pos.m	**Acquired Time**	3/14/2025 3:48:00 PM (UTC+03:00)
**IRM Calibration Status**	Success	**DA Method**	hcan.m
**Comment**			

**Sample Group**		**Info.**	
**Stream Name**	LC 1	**Method Version**	2025-0312-1046-26927
**Override DA Method Version**		**Data File Version**	2025-0314-1247-57662
**Acquisition Workstation**	DESKTOP-L73MD3C	**DA Workstation**	DESKTOP-L73MD3C
**Acquisition Time (Local)**	3/14/2025 3:48:00 PM (UTC+03:00)	**Acquisition SW Version**	6200 series TOF/6500 series Q-TOF (11.0.203.0)
**QTOF Driver Version**	11.00.00	**QTOF Firmware Version**	15.851
**Tune Mass Range Max.**	3200		

Chromatograms

Fragmentor Voltage 90 **Collision Energy** 0 **Ionization Mode** ESI

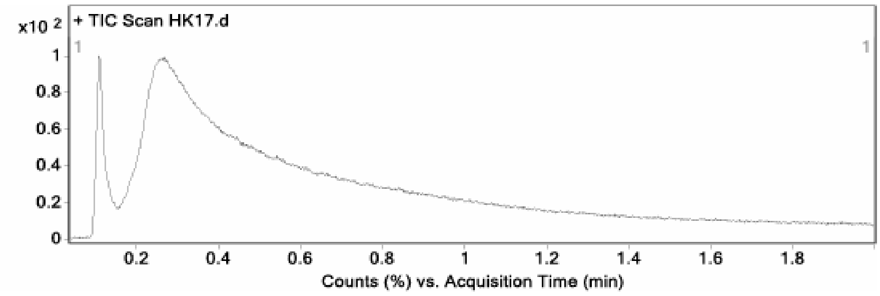

Spectra

Fragmentor Voltage 90 **Collision Energy** 0 **Ionization Mode** ESI

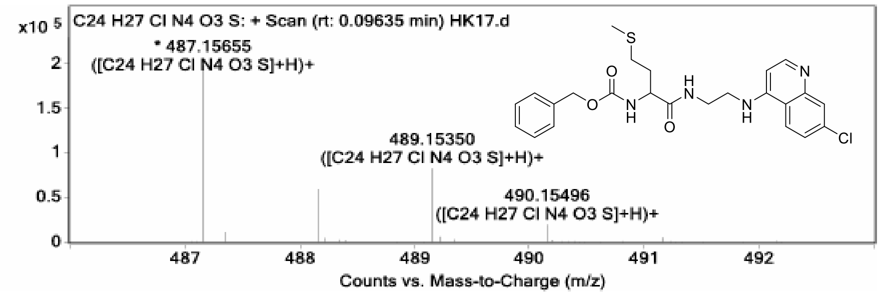

Figure S19: HRMS of Benzyl (1-((2-((7-chloroquinolin-4-yl)amino)ethyl)amino)-4-(methylthio)-1-oxobutan-2-yl)carbamate (**4d**)

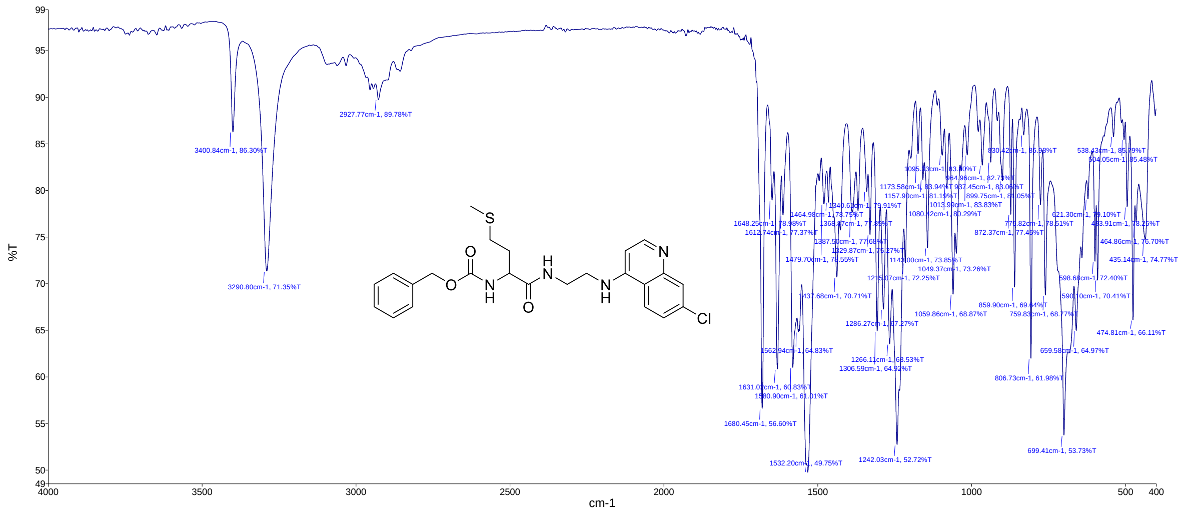

Figure S20: FTIR of Benzyl (1-((2-((7-chloroquinolin-4-yl)amino)ethyl)amino)-4-(methylthio)-1-oxobutan-2-yl)carbamate (**4d**)

S1.7**:** Spectra data of Benzyl (1-((3-((7-chloroquinolin-4-yl)amino)propyl)amino)-4-(methylthio)-1-oxobutan-2-yl)carbamate (**4e**)

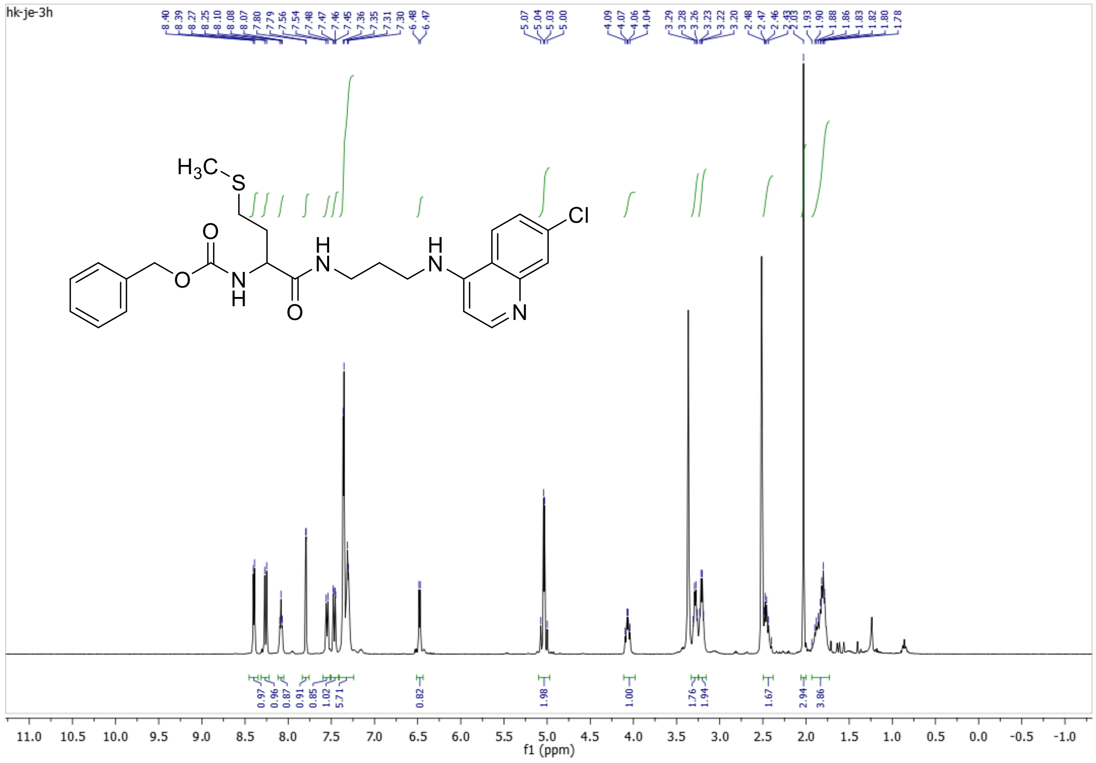

Figure S21: ^{1}H NMR Spectra of Benzyl (1-((3-((7-chloroquinolin-4-yl)amino)propyl)amino)-4-(methylthio)-1-oxobutan-2-yl)carbamate (**4e**)

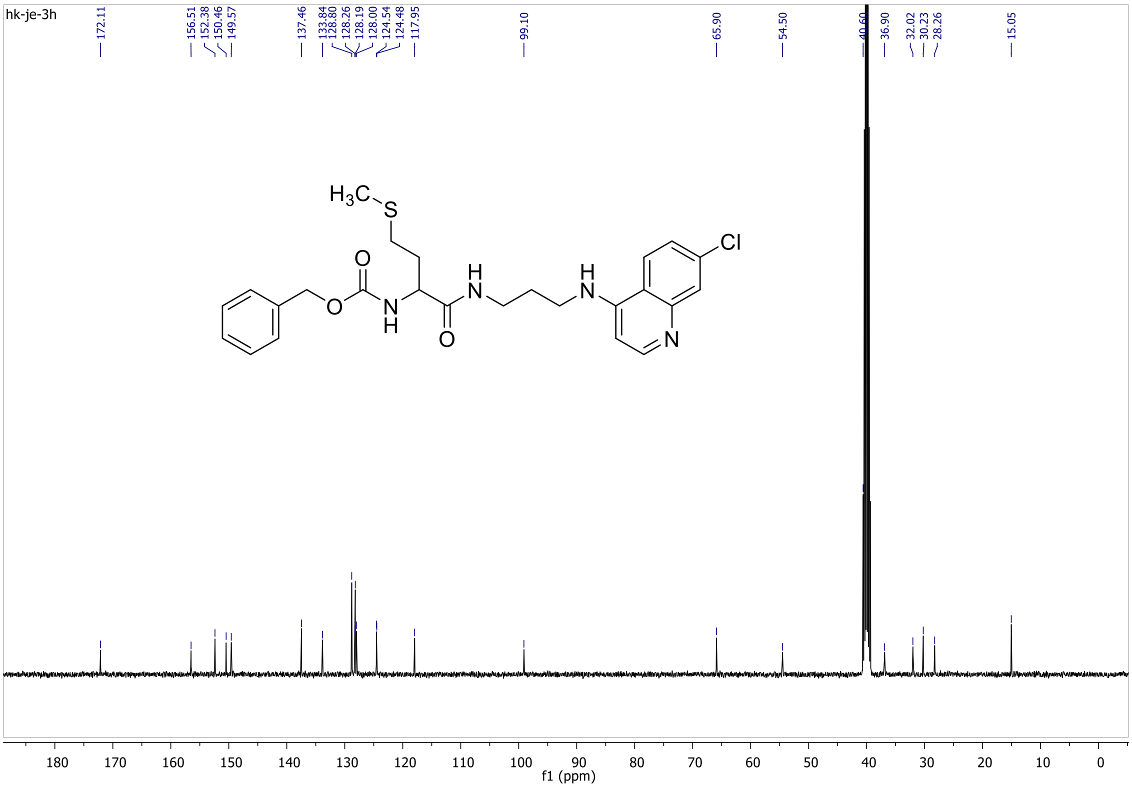

Figure S22: ^{13}C NMR Spectra of Benzyl (1-((3-((7-chloroquinolin-4-yl)amino)propyl)amino)-4-(methylthio)-1-oxobutan-2-yl)carbamate (**4e**)

Qualitative Analysis Report

**Data Filename**	HK19.d	**Sample Name**	HK19
**Sample Type**	Sample	**Position**	P1-C1
**Instrument Name**	6530B LC Q-TOF	**User Name**	OQADMIN (oqadmin)
**Acq Method**	ESI_Pos.m	**Acquired Time**	3/14/2025 3:53:28 PM (UTC+03:00)
**IRM Calibration Status**	Success	**DA Method**	hcan.m
**Comment**			

**Sample Group**		**Info.**	
**Stream Name**	LC 1	**Method Version**	2025-0312-1046-26927
**Override DA Method Version**		**Data File Version**	2025-0314-1253-24858
**Acquisition Workstation**	DESKTOP-L73MD3C	**DA Workstation**	DESKTOP-L73MD3C
**Acquisition Time (Local)**	3/14/2025 3:53:28 PM (UTC+03:00)	**Acquisition SW Version**	6200 series TOF/6500 series Q-TOF (11.0.203.0)
**QTOF Driver Version**	11.00.00	**QTOF Firmware Version**	15.851
**Tune Mass Range Max.**	3200		

Chromatograms

Fragmentor Voltage 90 **Collision Energy** 0 **Ionization Mode** ESI

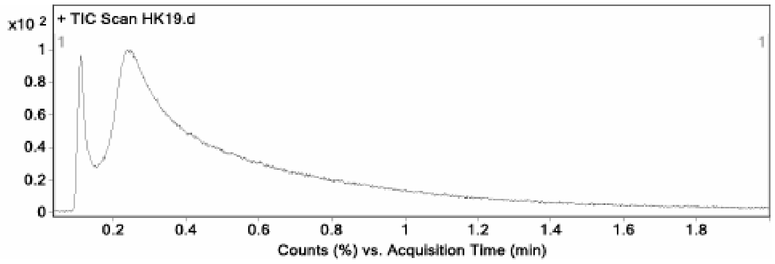

Spectra

Fragmentor Voltage 90 **Collision Energy** 0 **Ionization Mode** ESI

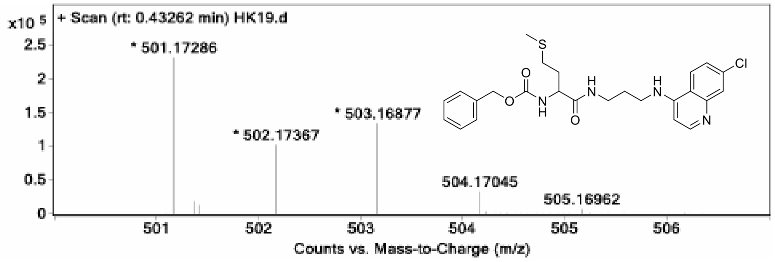

[illegible]

Figure S23: HRMS of Benzyl (1-((3-((7-chloroquinolin-4-yl)amino)propyl)amino)-4-(methylthio)-1-oxobutan-2-yl)carbamate (**4e**)

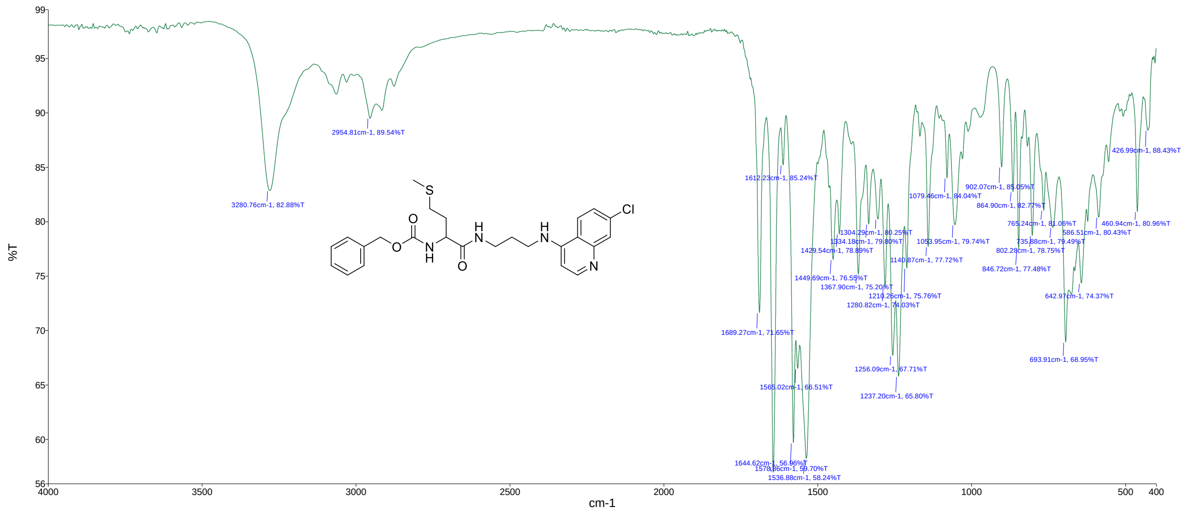

Figure S24: FTIR of Benzyl (1-((3-((7-chloroquinolin-4-yl)amino)propyl)amino)-4-(methylthio)-1-oxobutan-2-yl)carbamate (**4e**).

S1.8: Spectra data of *tert*-butyl (1-((2-((7-chloroquinolin-4-yl)amino)ethyl)amino)-3-methyl-1-oxobutan-2-yl)carbamate (**4f**)

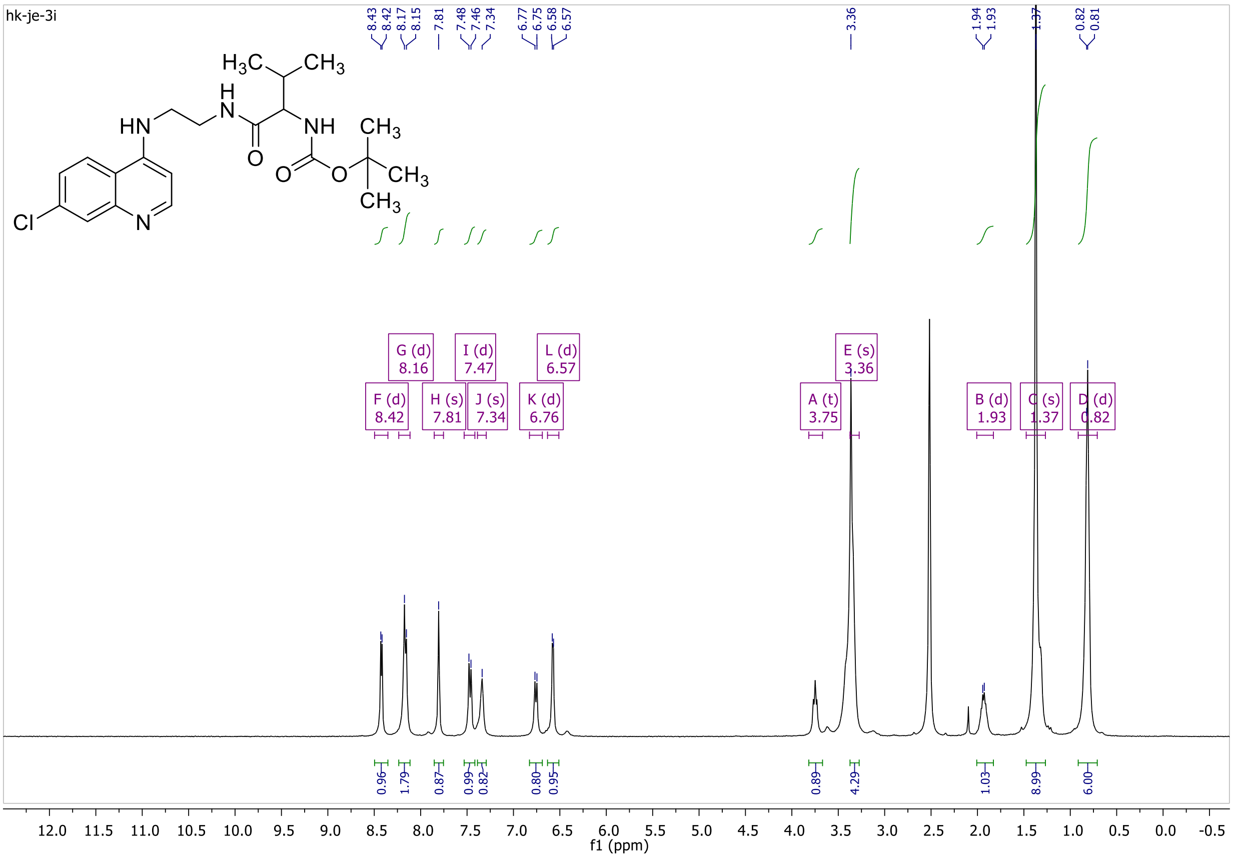

Figure S25: ^{1}H NMR Spectra of *tert*-butyl (1-((2-((7-chloroquinolin-4-yl)amino)ethyl)amino)-3-methyl-1-oxobutan-2-yl)carbamate (**4f**)

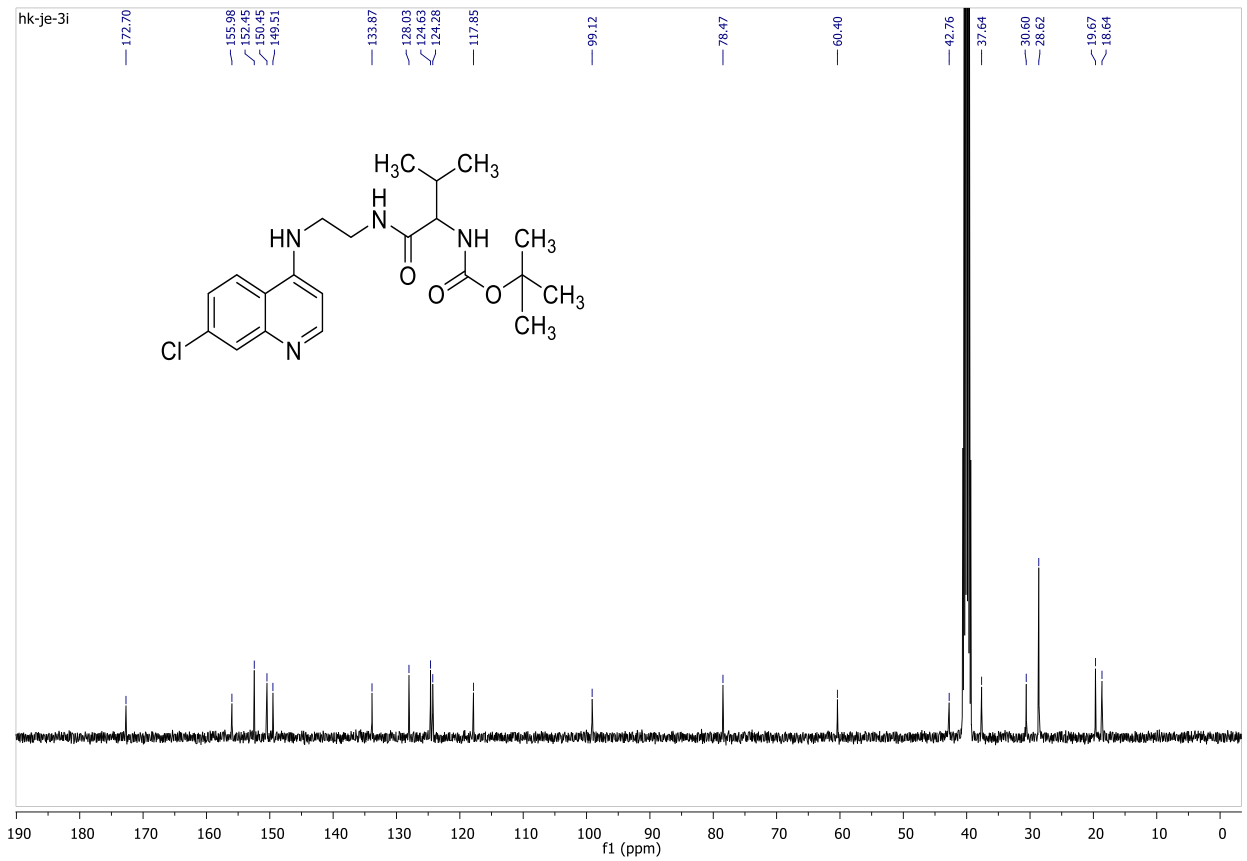

Figure S26: ^{13}C NMR Spectra of *tert*-butyl (1-((2-((7-chloroquinolin-4-yl)amino)ethyl)amino)-3-methyl-1-oxobutan-2-yl)carbamate (**4f**)

Qualitative Analysis Report

**Data Filename**	HK20.d	**Sample Name**	HK20
**Sample Type**	Sample	**Position**	P1-C2
**Instrument Name**	6530B LC Q-TOF	**User Name**	OQADMIN (oqadmin)
**Acq Method**	ESI_Pos.m	**Acquired Time**	3/14/2025 3:56:13 PM (UTC+03:00)
**IRM Calibration Status**	Success	**DA Method**	hcan.m
**Comment**			

**Sample Group**		**Info.**	
**Stream Name**	LC 1	**Method Version**	2025-0312-1046-26927
**Override DA Method Version**		**Data File Version**	2025-0314-1256-07567
**Acquisition Workstation**	DESKTOP-L73MD3C	**DA Workstation**	DESKTOP-L73MD3C
**Acquisition Time (Local)**	3/14/2025 3:56:13 PM (UTC+03:00)	**Acquisition SW Version**	6200 series TOF/6500 series Q-TOF (11.0.203.0)
**QTOF Driver Version**	11.00.00	**QTOF Firmware Version**	15.851
**Tune Mass Range Max.**	3200		

Chromatograms

Fragmentor Voltage 90 **Collision Energy** 0 **Ionization Mode** ESI

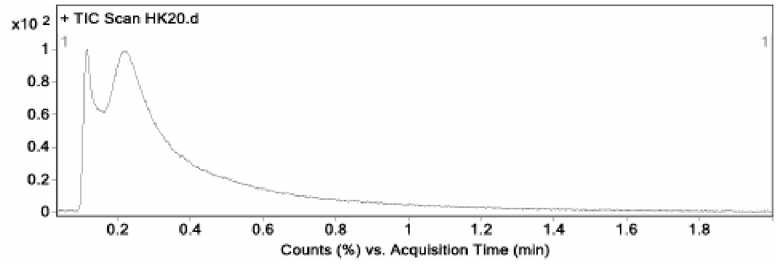

Spectra

Fragmentor Voltage	Collision Energy	Ionization Mode
90	0	ESI

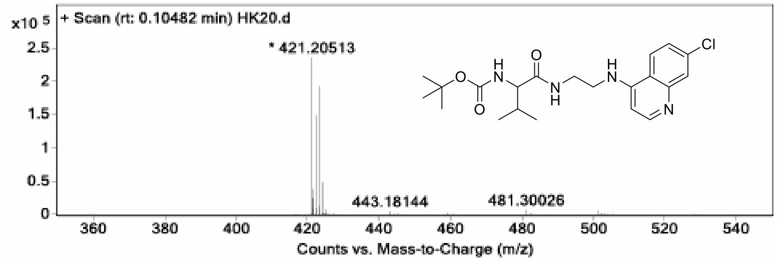

Peak List

m/z	z	Abund

Figure S27: HRMS of *tert*-butyl (1-((2-((7-chloroquinolin-4-yl)amino)ethyl)amino)-3-methyl-1-oxobutan-2-yl)carbamate (**4f**)

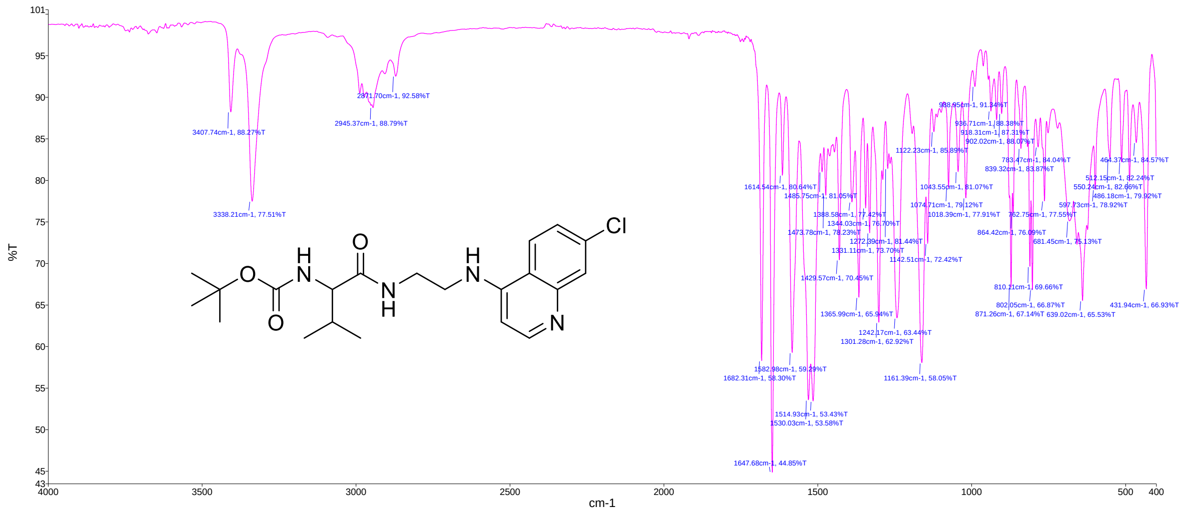

Figure S28: FTIR of *tert*-butyl (1-((2-((7-chloroquinolin-4-yl)amino)ethyl)amino)-3-methyl-1-oxobutan-2-yl)carbamate (**4f**)

S1.9: Spectra data of tert-butyl (1-((3-((7-chloroquinolin-4-yl)amino)propyl)amino)-3-methyl-1-oxobutan-2-yl)carbamate (**4g**)

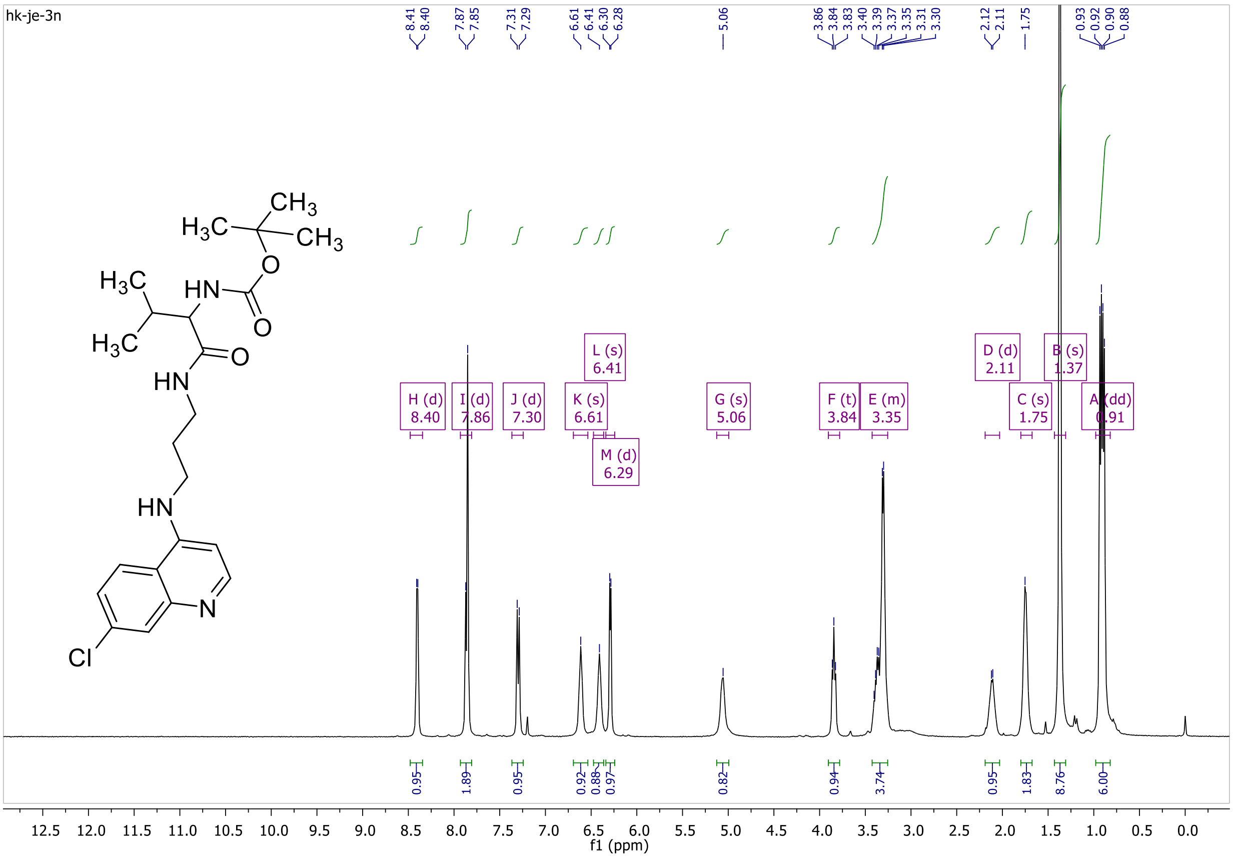

Figure S29: ^{1}H NMR Spectrum of tert-butyl (1-((3-((7-chloroquinolin-4-yl)amino)propyl)amino)-3-methyl-1-oxobutan-2-yl)carbamate (**4g**)

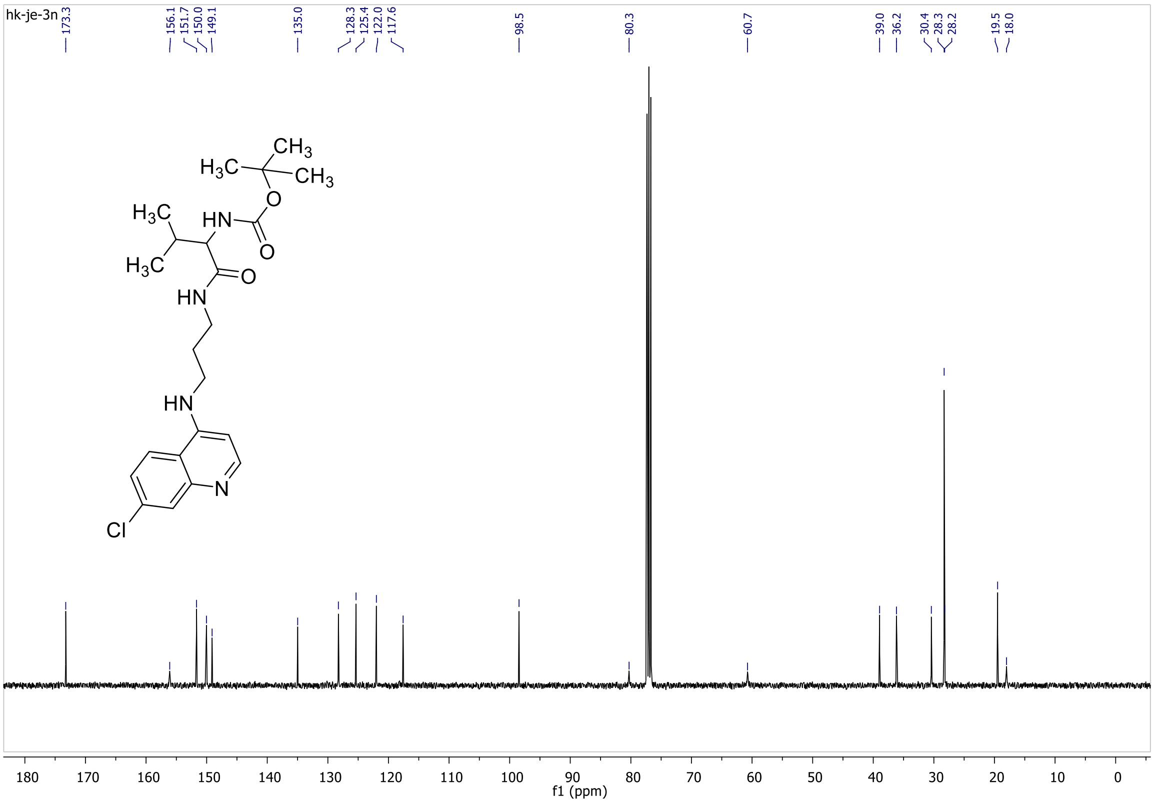

Figure S30: ^{13}C NMR Spectrum of tert-butyl (1-((3-((7-chloroquinolin-4-yl)amino)propyl)amino)-3-methyl-1-oxobutan-2-yl)carbamate (**4g**)

Qualitative Analysis Report

**Data Filename**	HK23.d	**Sample Name**	HK23
**Sample Type**	Sample	**Position**	P1-C5
**Instrument Name**	6530B LC Q-TOF	**User Name**	OQADMIN (oqadmin)
**Acq Method**	ESI_Pos.m	**Acquired Time**	5/6/2025 5:25:13 PM (UTC+03:00)
**IRM Calibration Status**	Success	**DA Method**	hcan.m
**Comment**			

**Sample Group**		**Info.**	
**Stream Name**	LC 1	**Method Version**	2025-0505-0809-01740
**Override DA Method Version**		**Data File Version**	2025-0506-1425-08397
**Acquisition Workstation**	DESKTOP-L73MD3C	**DA Workstation**	DESKTOP-L73MD3C
**Acquisition Time (Local)**	5/6/2025 5:25:13 PM (UTC+03:00)	**Acquisition SW Version**	6200 series TOF/6500 series Q-TOF (11.0.203.0)
**QTOF Driver Version**	11.00.00	**QTOF Firmware Version**	15.851
**Tune Mass Range Max.**	3200		

Chromatograms

Fragmentor Voltage 90 **Collision Energy** 0 **Ionization Mode** ESI

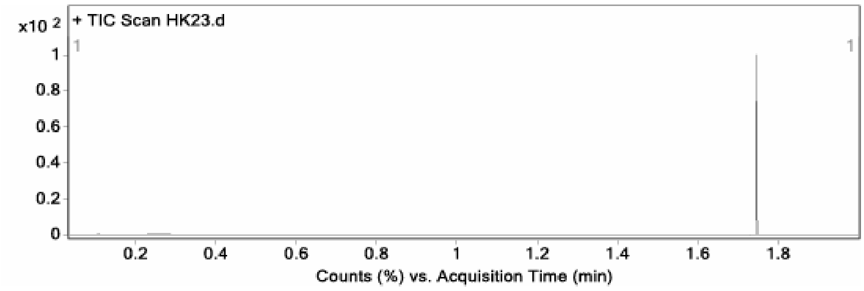

Spectra

Fragmentor Voltage 90 **Collision Energy** 0 **Ionization Mode** ESI

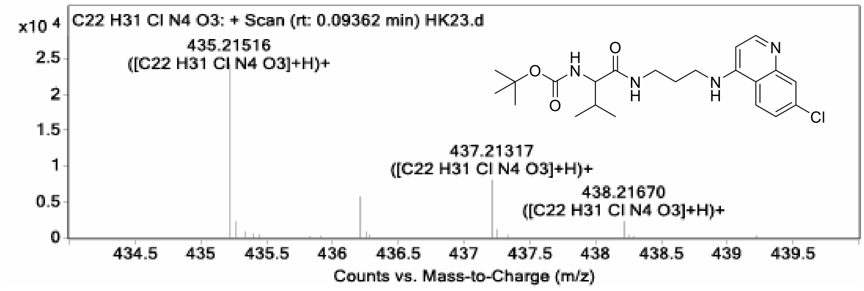

Figure S31:HRMS of *tert*-butyl (1-((3-((7-chloroquinolin-4-yl)amino)propyl)amino)-3-methyl-1-oxobutan-2-yl) carbamate (**4g**)

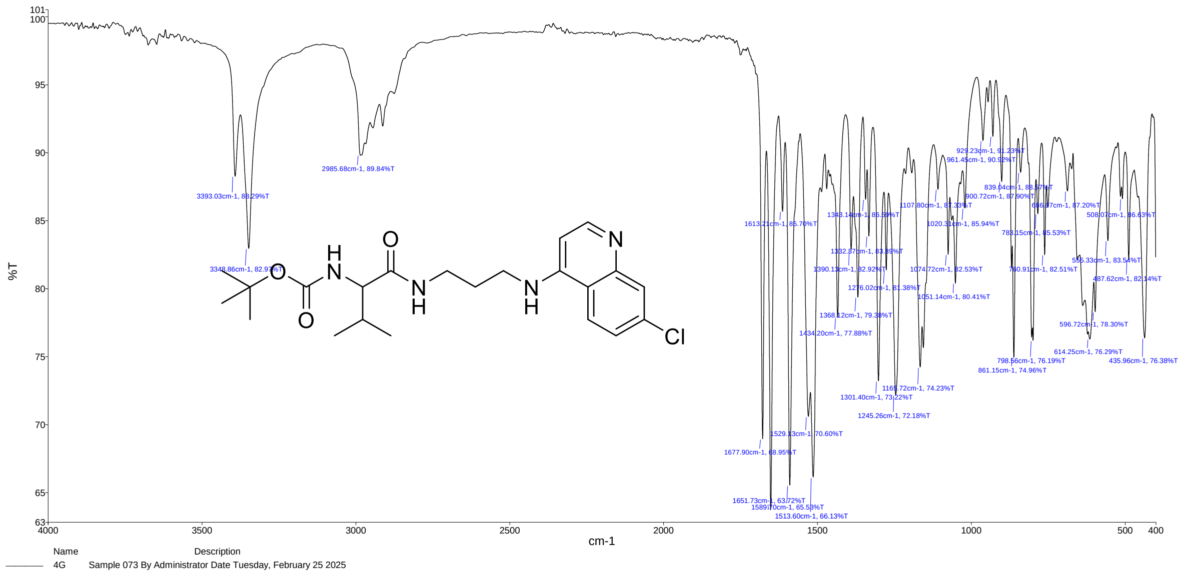

Figure S32: FTIR of *tert*-butyl (1-((3-((7-chloroquinolin-4-yl)amino)propyl)amino)-3-methyl-1-oxobutan-2-yl)carbamate (**4g**)

S1.10: Spectra data of tert-butyl (1-((2-((7-chloroquinolin-4-yl)amino)ethyl)amino)-4-(methylthio)-1-oxobutan-2-yl)carbamate (**4h**)

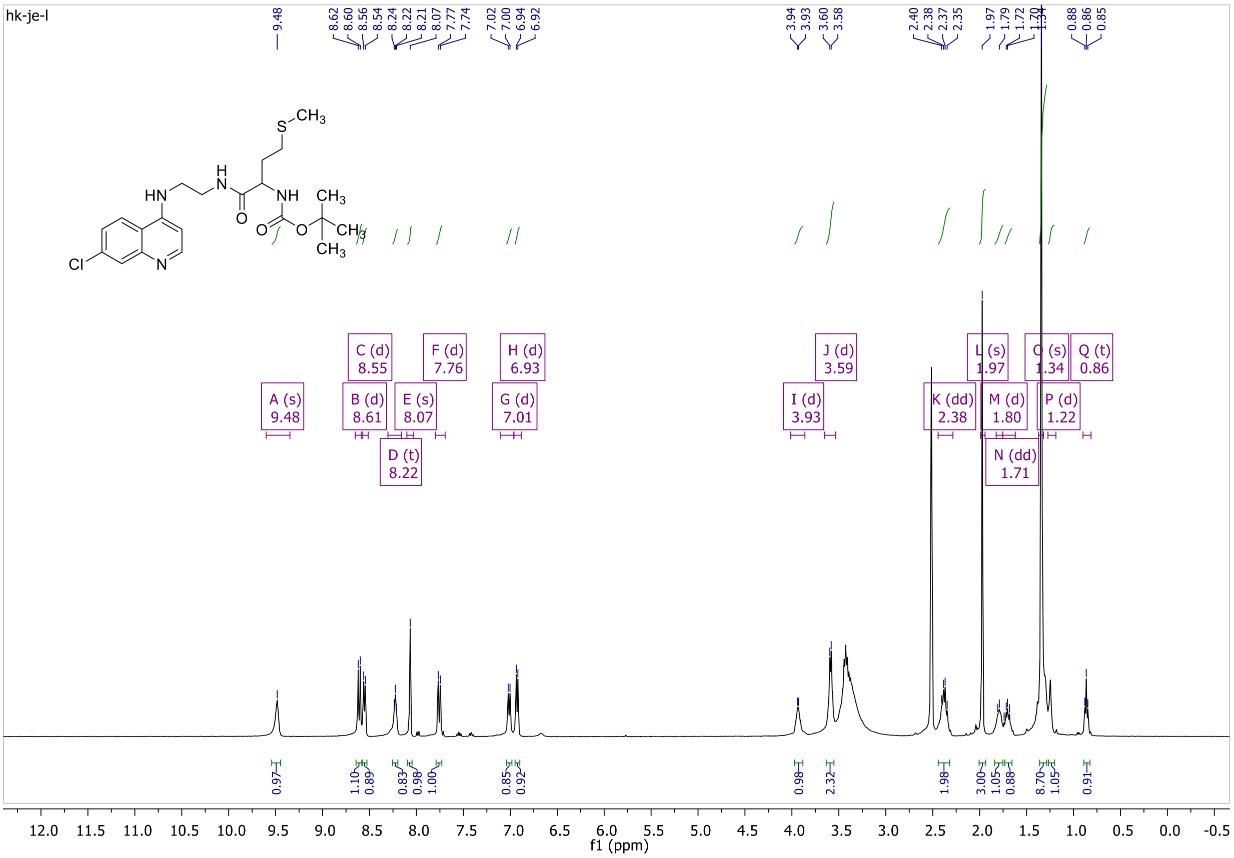

Figure S33: ^{1}H NMR Spectrum of tert-butyl (1-((2-((7-chloroquinolin-4-yl)amino)ethyl)amino)-4-(methylthio)-1-oxobutan-2-yl)carbamate (**4h**)

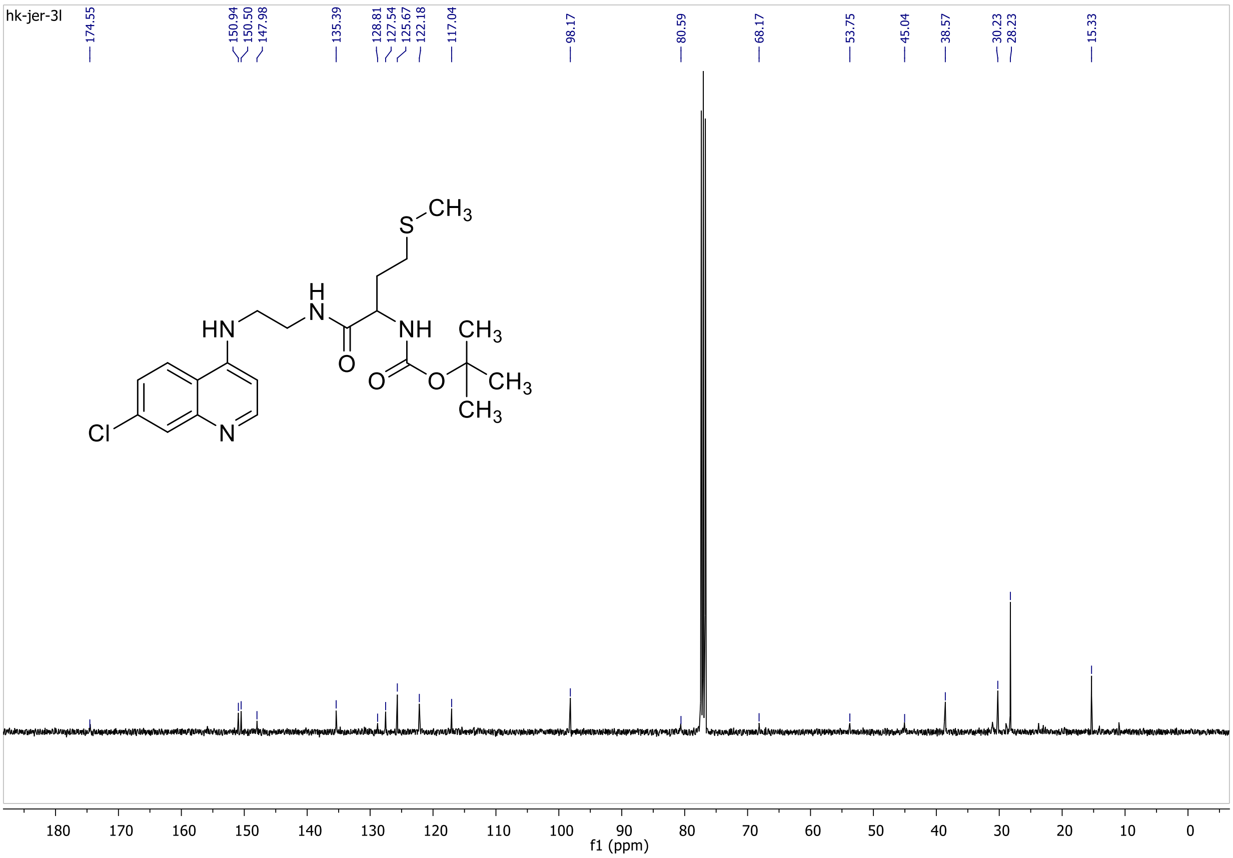

Figure S34: ^{13}C NMR Spectrum of tert-butyl (1-((2-((7-chloroquinolin-4-yl)amino)ethyl)amino)-4-(methylthio)-1-oxobutan-2-yl)carbamate (**4h**)

Qualitative Analysis Report

**Data Filename**	HK22.d	**Sample Name**	HK22
**Sample Type**	Sample	**Position**	P1-C4
**Instrument Name**	6530B LC Q-TOF	**User Name**	OQADMIN (oqadmin)
**Acq Method**	ESI_Pos.m	**Acquired Time**	5/6/2025 5:22:29 PM (UTC+03:00)
**IRM Calibration Status**	Success	**DA Method**	hcan.m
**Comment**			

**Sample Group**		**Info.**	
**Stream Name**	LC 1	**Method Version**	2025-0505-0809-01740
**Override DA Method Version**		**Data File Version**	2025-0506-1422-25084
**Acquisition Workstation**	DESKTOP-L73MD3C	**DA Workstation**	DESKTOP-L73MD3C
**Acquisition Time (Local)**	5/6/2025 5:22:29 PM (UTC+03:00)	**Acquisition SW Version**	6200 series TOF/6500 series Q-TOF (11.0.203.0)
**QTOF Driver Version**	11.00.00	**QTOF Firmware Version**	15.851
**Tune Mass Range Max.**	3200		

Chromatograms

Fragmentor Voltage	Collision Energy	Ionization Mode
90	0	ESI

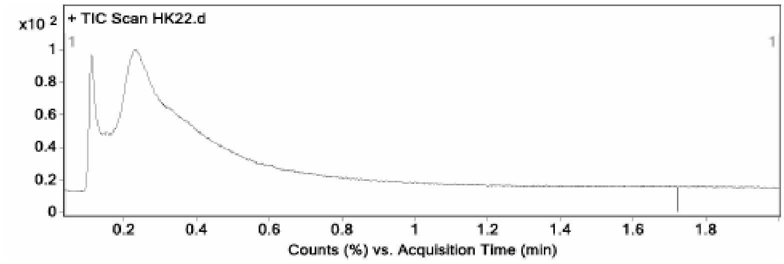

Spectra

Fragmentor Voltage	Collision Energy	Ionization Mode
90	0	ESI

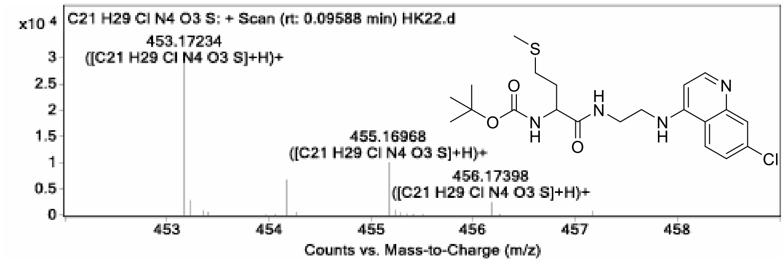

Figure S35: HRMS of *tert*-butyl (1-((2-((7-chloroquinolin-4-yl)amino)ethyl)amino)-4-(methylthio)-1-oxobutan-2-yl)carbamate (**4h**)

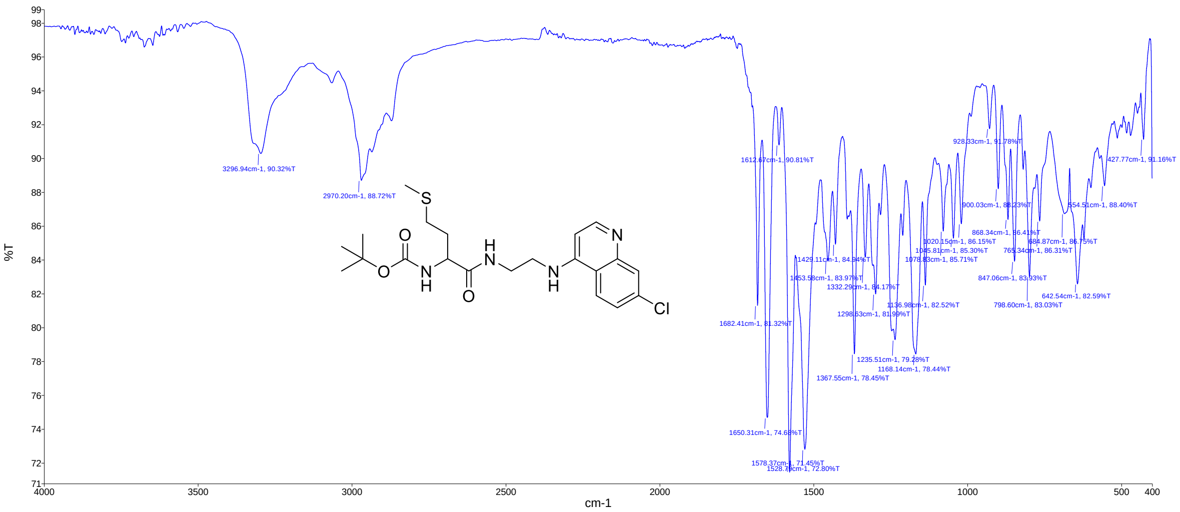

Figure S36: FTIR of *tert*-butyl (1-((2-((7-chloroquinolin-4-yl)amino)ethyl)amino)-4-(methylthio)-1-oxobutan-2-yl)carbamate (**4h**)

S1.11: Spectra data of 2-amino-*N*-(3-((7-chloroquinolin-4-yl)amino)propyl)-3-methylbutanamide (**5a**)

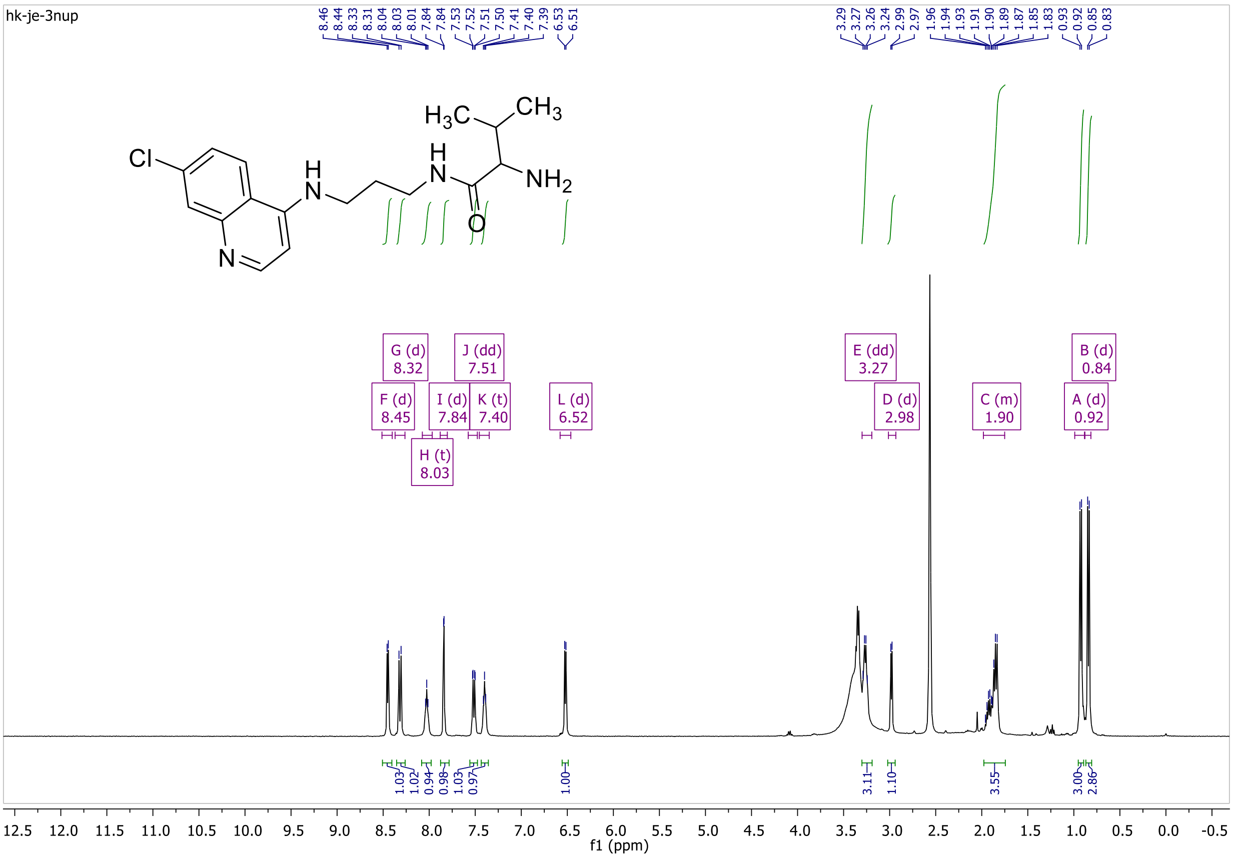

Figure S37: ^{1}H NMR Spectrum of 2-amino-*N*-(3-((7-chloroquinolin-4-yl)amino)propyl)-3-methylbutanamide (**5a**)

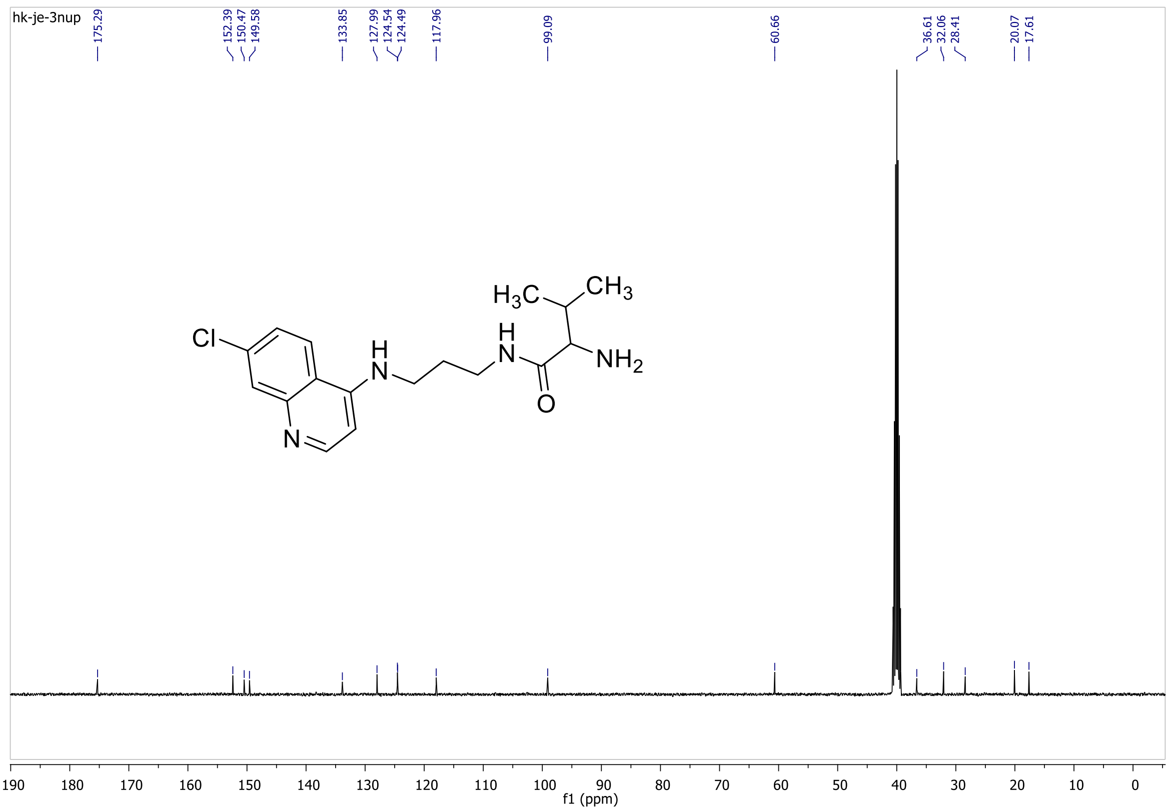

Figure S38: ^{13}C NMR Spectrum of 2-amino-*N*-(3-((7-chloroquinolin-4-yl)amino)propyl)-3-methylbutanamide (**5a**)

Qualitative Analysis Report

**Data Filename**	HK24.d	**Sample Name**	HK24
**Sample Type**	Sample	**Position**	P1-C6
**Instrument Name**	6530B LC Q-TOF	**User Name**	OQADMIN (oqadmin)
**Acq Method**	ESI_Pos.m	**Acquired Time**	5/6/2025 5:27:57 PM (UTC+03:00)
**IRM Calibration Status**	Success	**DA Method**	hcan.m
**Comment**			

**Sample Group**		**Info.**	
**Stream Name**	LC 1	**Method Version**	2025-0505-0809-01740
**Override DA Method Version**		**Data File Version**	2025-0506-1427-52541
**Acquisition Workstation**	DESKTOP-L73MD3C	**DA Workstation**	DESKTOP-L73MD3C
**Acquisition Time (Local)**	5/6/2025 5:27:57 PM (UTC+03:00)	**Acquisition SW Version**	6200 series TOF/6500 series Q-TOF (11.0.203.0)
**QTOF Driver Version**	11.00.00	**QTOF Firmware Version**	15.851
**Tune Mass Range Max.**	3200		

Chromatograms

Fragmentor Voltage 90 **Collision Energy** 0 **Ionization Mode** ESI

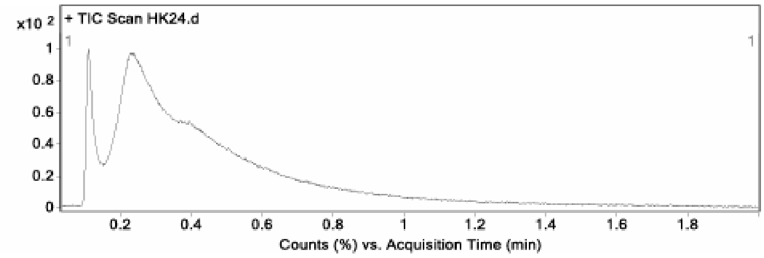

Spectra

Fragmentor Voltage 90 **Collision Energy** 0 **Ionization Mode** ESI

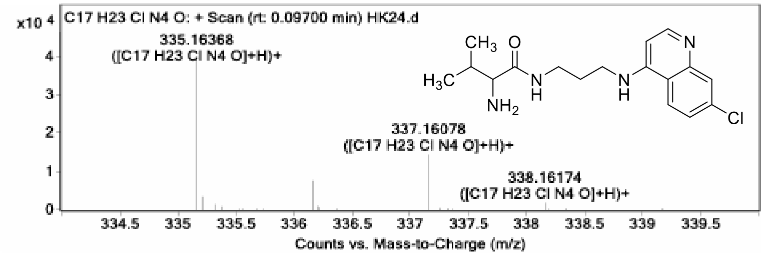

Figure S39: HRMS of 2-amino-*N*-(3-((7-chloroquinolin-4-yl)amino)propyl)-3-methylbutanamide (**5a**)

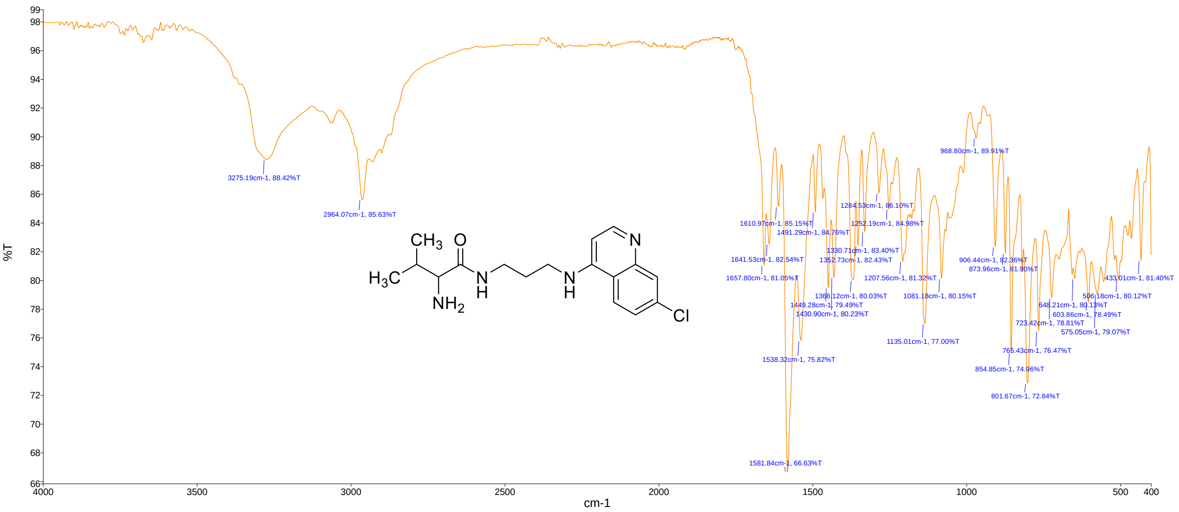

Figure S40: FTIR of 2-amino-N-(3-((7-chloroquinolin-4-yl)amino)propyl)-3-methylbutanamide (**5a**)

S1.12: Spectra data of 2-amino-*N*-(2-((7-chloroquinolin-4-yl)amino)ethyl)-4-(methylthio)butanamide (**5b**)

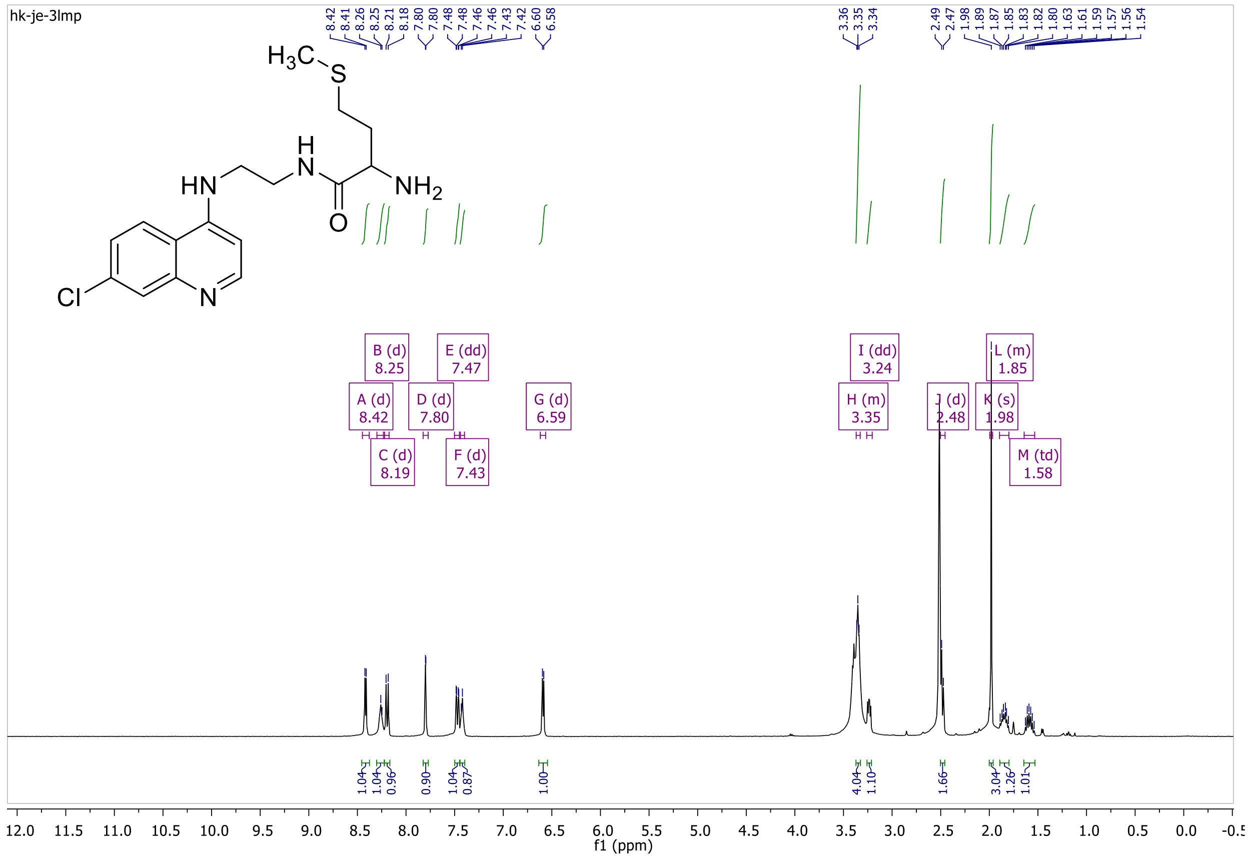

Figure S41: ^{1}H NMR Spectrum of 2-amino-*N*-(2-((7-chloroquinolin-4-yl)amino)ethyl)-4-(methylthio)butanamide (**5b**)

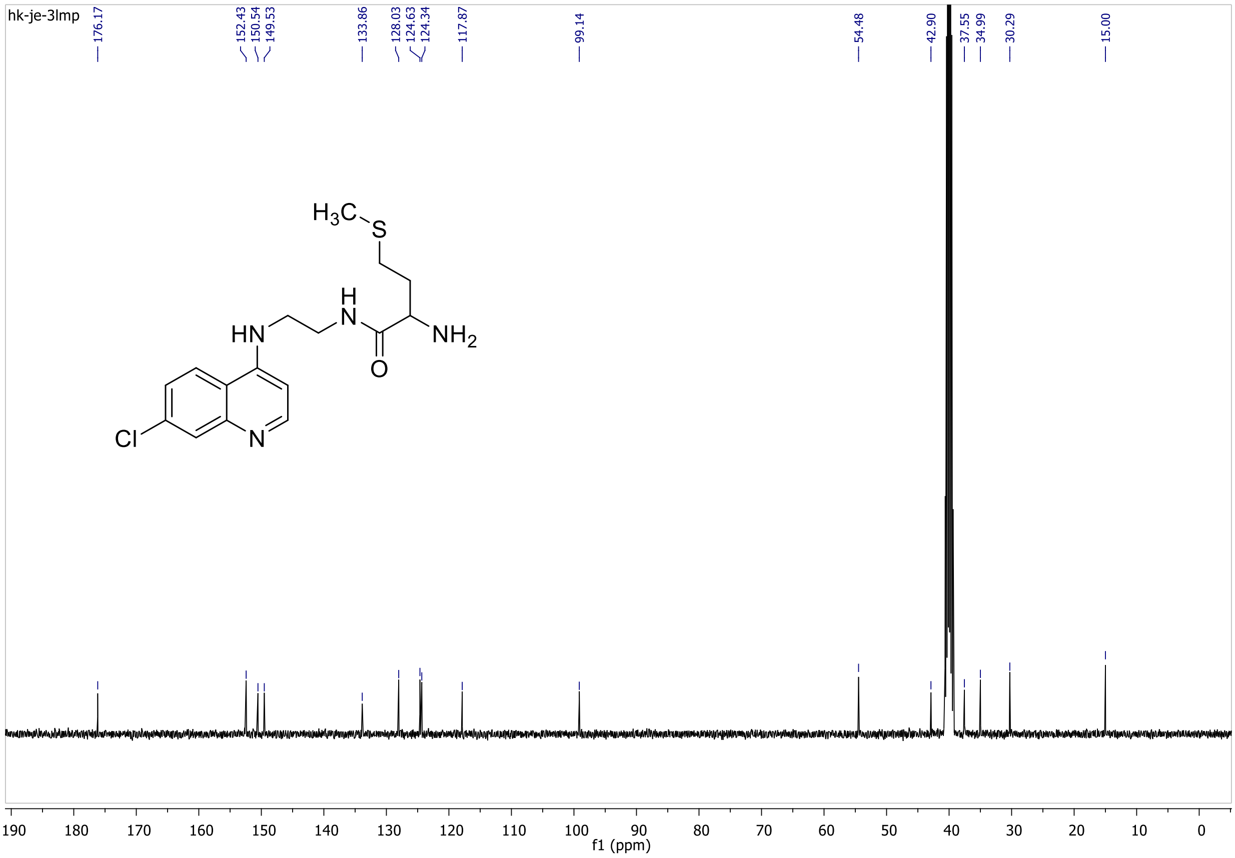

Figure S42: ^{13}C NMR Spectrum of 2-amino-*N*-(2-((7-chloroquinolin-4-yl)amino)ethyl)-4-(methylthio)butanamide (**5b**)

Qualitative Analysis Report

**Data Filename**	HK25.d	**Sample Name**	HK25
**Sample Type**	Sample	**Position**	P1-C7
**Instrument Name**	6530B LC Q-TOF	**User Name**	OQADMIN (oqadmin)
**Acq Method**	ESI_Pos.m	**Acquired Time**	5/6/2025 5:30:41 PM (UTC+03:00)
**IRM Calibration Status**	Success	**DA Method**	hcan.m
**Comment**			

**Sample Group**		**Info.**	
**Stream Name**	LC 1	**Method Version**	2025-0505-0809-01740
**Override DA Method Version**		**Data File Version**	2025-0506-1430-37064
**Acquisition Workstation**	DESKTOP-L73MD3C	**DA Workstation**	DESKTOP-L73MD3C
**Acquisition Time (Local)**	5/6/2025 5:30:41 PM (UTC+03:00)	**Acquisition SW Version**	6200 series TOF/6500 series Q-TOF (11.0.203.0)
**QTOF Driver Version**	11.00.00	**QTOF Firmware Version**	15.851
**Tune Mass Range Max.**	3200		

Chromatograms

Fragmentor Voltage 90 **Collision Energy** 0 **Ionization Mode** ESI

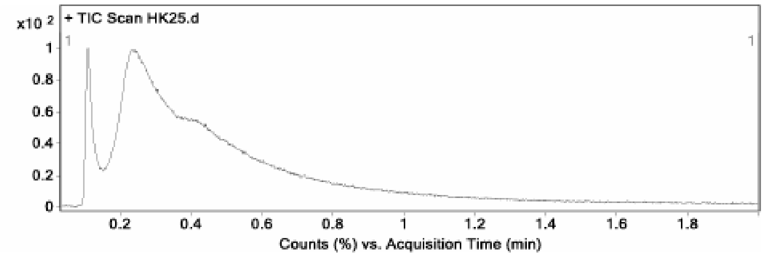

Spectra

**Fragmentor Voltage**	**Collision Energy**	**Ionization Mode**
90	0	ESI

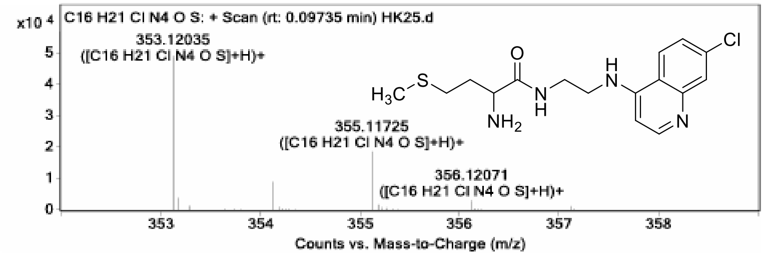

Peak List

Figure S43: HRMS of 2-amino-*N*-(2-((7-chloroquinolin-4-yl)amino)ethyl)-4-(methylthio)butanamide (**5b**)

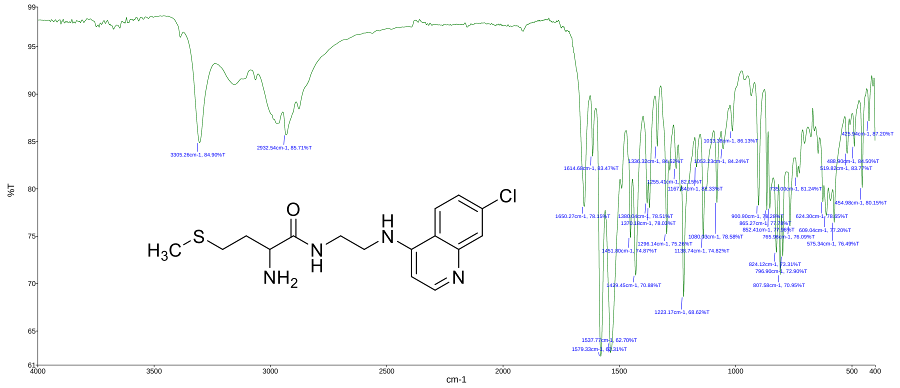

Figure S44: FTIR of 2-amino-*N*-(2-((7-chloroquinolin-4-yl)amino)ethyl)-4-(methylthio)butanamide (**5b**)
